# Increased Postnatal Cardiac Hyperplasia Precedes Cardiomyocyte Hypertrophy in a Model of Hypertrophic Cardiomyopathy

**DOI:** 10.3389/fphys.2017.00414

**Published:** 2017-06-14

**Authors:** Emily T. Farrell, Adrian C. Grimes, Willem J. de Lange, Annie E. Armstrong, J. Carter Ralphe

**Affiliations:** ^1^Department of Pediatrics, University of Wisconsin School of Medicine and Public HealthMadison, WI, United States; ^2^Department of Medicine, University of Wisconsin School of Medicine and Public HealthMadison, WI, United States

**Keywords:** hypertrophic cardiomyopathy, HCM, myosin binding protein C, MyBP-C, hyperplasia, proliferation, cell cycling

## Abstract

**Rationale:** Hypertrophic cardiomyopathy (HCM) occurs in ~0.5% of the population and is a leading cause of sudden cardiac death (SCD) in young adults. Cardiomyocyte hypertrophy has been the accepted mechanism for cardiac enlargement in HCM, but the early signaling responsible for initiating hypertrophy is poorly understood. Mutations in cardiac myosin binding protein C (*MYBPC3*) are among the most common HCM-causing mutations. Ablation of *Mybpc3* in an HCM mouse model (cMyBP-C^−/−^) rapidly leads to cardiomegaly by postnatal day (PND) 9, though hearts are indistinguishable from wild-type (WT) at birth. This model provides a unique opportunity to explore early processes involved in the dramatic postnatal transition to hypertrophy.

**Methods and Results:** We performed microarray analysis, echocardiography, qPCR, immunohistochemistry (IHC), and isolated cardiomyocyte measurements to characterize the perinatal cMyBP-C^−/−^ phenotype before and after overt hypertrophy. cMyBP-C^−/−^ hearts showed elevated cell cycling at PND1 that transitioned to hypertrophy by PND9. An expanded time course revealed that increased cardiomyocyte cycling was associated with elevated heart weight to body weight ratios prior to cellular hypertrophy, suggesting that cell cycling resulted in cardiomyocyte proliferation. Animals heterozygous for the cMyBP-C deletion trended in the direction of the homozygous null, yet did not show increased heart size by PND9.

**Conclusions:** Results indicate that altered regulation of the cell cycling pathway and elevated proliferation precedes hypertrophy in the cMyBP-C^−/−^ HCM model, and suggests that increased cardiomyocyte number contributes to increased heart size in cMyBP-C^−/−^ mice. This pre-hypertrophic period may reflect a unique time during which the commitment to HCM is determined and disease severity is influenced.

## Introduction

Hypertrophic cardiomyopathy (HCM) is a prevalent cause of heart failure in adults and a leading cause of sudden cardiac death (SCD) in apparently healthy young individuals (Fananapazir and Epstein, 1991). It is a primary cardiac disease inherited in autosomal dominant fashion and estimated to affect 1 in 200 individuals (Spirito et al., 1997; Semsarian et al., 2015). The features of HCM include left ventricular hypertrophy, myocardial disarray, fibrosis, diastolic dysfunction, and an increased risk of both SCD and congestive heart failure (Fananapazir and Epstein, 1994; Davies and McKenna, 1995). HCM has long been regarded as a disease of late adolescence and adulthood. Involvement in neonates and young children is relatively rare, but early-onset HCM is typically quite severe (Van Driest et al., 2004; Xin et al., 2007; Zahka et al., 2008). At least 14 HCM-causative genes have been identified, with the majority of genotype-positive cases of familial HCM resulting from mutations in the genes for either β-myosin heavy chain (*MYH7*) or *MYBPC3*, which encodes cardiac myosin binding protein C (cMyBP-C; Richard et al., 2003; Olivotto et al., 2008; Alfares et al., 2015). cMyBP-C is a thick filament regulatory protein that binds myosin (Okagaki et al., 1993; Gruen and Gautel, 1999), titin (Labeit et al., 1992), and actin (Razumova et al., 2006), and functions as an important determinant of cardiac contractile reserve (Tong et al., 2008; de Lange et al., 2013). Nearly 200 disease-associated mutations in cMyBP-C are described and include missense mutations and early truncations (Harris et al., 2011). While it is recognized that the majority of humans with genotype-positive HCM are heterozygotes, homozygosity, and compound heterozygosity do exist and are associated with early-onset and typically more severe HCM (Xin et al., 2007; Zahka et al., 2008; Ripoll Vera et al., 2010).

We and others previously reported a mouse model of HCM in which cMyBP-C is genetically ablated (Harris et al., 2002; Korte et al., 2003; Stelzer et al., 2006; de Lange et al., 2011, 2013). This model recapitulates several important aspects of human HCM with both sarcomere dysfunction and a pronounced and fully penetrant phenotype described as hypertrophic (Harris et al., 2002; Korte et al., 2003; Stelzer et al., 2006; Moss et al., 2015). However, a remarkable characteristic of this germline ablation of an important structural and regulatory protein is that it appears to have negligible impact on embryonic cardiac development, while producing a severe hypertrophic phenotype over a very rapid time frame after birth (de Lange et al., 2013). The heart of the postnatal day (PND) 1 cMyBP-C^−/−^ mouse is indistinguishable from WT, but significantly and rapidly enlarges over the first 9 days of life—as demonstrated by histology and heart weight (HW) to body weight (BW) ratios (Harris et al., 2002; de Lange et al., 2011). The mechanisms involved in the rapid development of HCM during the perinatal transition have not been previously explored in the cMyBP-C^−/−^ mouse. Our initial hypothesis was that hypertrophic signaling pathways would be differentially upregulated in the cMyBP-C^−/−^ myocardium at PND1, prior to the observed gross morphologic changes seen by PND9. Full characterization and improved understanding of the molecular events during this period could identify potential therapeutic targets to modify disease course.

In this study, we performed a microarray analysis comparing WT and cMyBP-C^−/−^ hearts at PND1 and PND9 to define differential gene regulation that may precede and contribute to the hypertrophic phenotype. Based on these results we then further examined the status of the perinatal heart in embryonic (E) day 18.5 to PND9 mice deficient in cMyBP-C to define the relative contribution of hyperplasia and/or hypertrophy in the development of the observed early cardiac enlargement. A recent study performed by Jiang et al. (2015) using a mouse model with a truncation mutation in *MYBPC3* (cMyBP-C^*t*/*t*^ mouse; McConnell et al., 1999; Sadayappan et al., 2006; Jiang et al., 2015), identified elevated cardiomyocyte proliferation extending through PND17 (Jiang et al., 2015). Unlike the cMyBP-C^−/−^ model, however, no cardiomyocyte hypertrophy was observed in the cMyBP-C^*t*/*t*^ mice through 5 weeks of age. Importantly, the cMyBP-C^−/−^ and cMyBP-C^*t*/*t*^ mice are fundamentally different, as the cMyBP-C^t/t^ mouse has been shown to produce protein, albeit at a low level (McConnell et al., 1999), whereas the cMyBP-C^−/−^ mouse is a true knockout with no protein expression. A comparison of the data from Jiang et al. with data presented here suggests that the two models develop cardiomyopathies through distinct cellular pathways, potentially highlighting a mutation-specific divergence in phenotype.

## Methods

### Animals

Heterozygous (cMyBP-C^+/−^) adults were derived from backcrosses of WT E129X1/SvJ mice (Taconic, Hudson, NY) with homozygous cMyBP-C^−/−^ animals previously generated on the E129X1/SvJ background (Harris et al., 2002). Subsequent breedings of cMyBP-C^+/−^ adults resulted in expected Mendelian frequencies (1:2:1) of WT (cMyBP-C^+/+^), heterozygous (cMyBP-C^+/−^) and homozygous knockout (cMyBP-C^−/−^) animals. Mice in this study were produced from MyBP-C heterozygote (MyBP-C^+/−^) crossings, with subsequent postmortem genotyping of pups. Littermate comparisons among genotypes were performed whenever possible.

Pups from these breedings were decapitated and intact hearts were harvested, rinsed in 1X PBS, and the blood ejected using blunt forceps. A post-mortem tail snip was taken from each mouse to allow subsequent genotyping and sex determination by PCR. Live postnatal day (PND) 1 and PND9 WT and cMyBP-C^−/−^ pups used for echocardiographic measurements were derived from cMyBP-C^−/−^ × cMyBP-C^−/−^ and WT × WT crosses. PND0 is defined here as the day of birth. This study was approved by the Animal Care and Use Committee of the School of Medicine and Public Health at the University of Wisconsin Madison in accordance with the Guide for the Care and Use of Laboratory Animals (National Institutes of Health publication no. 85-23, revised 1985).

### Generation of embryonic day (E)18.5 pups and extraction of hearts

cMyBP-C^+/−^ dams were bred to cMyBP-C^+/−^ males for 24 h, after which the males were removed from the cages to establish the timing of pregnancy. Pregnant dams were anesthetized using inhaled isoflurane on day 19 after the breeding date. The abdomens of the dams were opened and the uterine horns containing the embryos were placed in a petri dish with 1X PBS. Embryos were removed from the uterine horn and embryonic sacs, and weighed prior to isolation of each heart. Hearts were rinsed in 1X PBS, weighed and snap frozen in liquid nitrogen.

### Routine histology

PND1 and PND9 mouse hearts harvested as described were fixed and embedded in paraffin according to standard histological procedures detailed in Supplementary Methods. Sections were then stained with hematoxylin and eosin (H&E) using SelecTec reagents and protocols (Surgipath) for general assessment of tissue structure, or by commercially available Masson's Trichrome or Elastic Trichrome stains (Sigma) to highlight elastic fibers and collagen, or by picrosirius red stain (Polysciences, Inc.) to highlight interstitial fibrosis.

### Transthoracic echocardiography on WT and cMyBPC^−/−^ mice

Hearts of conscious PND1 and anesthetized PND9 mice were imaged using a Vevo 770 ultrasonograph (Visual Sonics) with a 40-MHz transducer (Agilent Technologies). Details regarding anesthesia and animal preparation are available in Supplementary Methods. Two-dimensionally guided M-mode images were acquired at the tip of papillary muscles, and transmitral and aortic velocities measured using Doppler pulse wave imaging. End diastolic and systolic left ventricular (LV) diameter, as well as anterior wall (AW) and posterior wall (PW) thickness, were measured on-line from M-mode images using the leading edge to leading edge convention. All parameters were measured over at least three consecutive cardiac cycles and averaged. Heart rate was determined from at least three consecutive intervals from pulse wave Doppler tracings of the LV outflow tract. Ejection fraction was calculated by 100 × (LV volume diastole - LV volume systole)/LV volume diastole. Isovolumic relaxation time was measured as the time between closing of the aortic valve and the opening of the mitral value from pulse wave Doppler tracings of the LV outflow tract and mitral inflow region.

### Microarray hybridization and data analysis

The left ventricular free wall (LVFW) was dissected from the hearts of PND1 and PND9 pups generated from the breeding of heterozygous cMyBP-C^+/−^ adults, and snap frozen in liquid nitrogen. After PCR determination of genotype and sex, the LVFW of seven males from each genotype and age was used for extraction of total RNA as described in Supplementary Methods. Quantification, quality and integrity of RNA were determined with the NanoDrop 2000 (Thermo Scientific), Agilent BioAnalyzer and Agilent RNA Nano Chip (Agilent Technologies). Four hundred nanograms of RNA (and 400 ng of poly-A RNA control) was used for overnight *in-vitro* labeling with the Ambion GeneChip WT Expression kit following manufacturer's protocols. Following labeling, samples were purified/quantified on the NanoDrop 2000 and 10 μg of purified cRNA used for template input to generate single strand cDNA, 5.5 μg of which was then fragmented and subjected to end-terminus labeling using the Affymetrix WT terminal Labeling Kit (Affymetrix). Samples were hybridized to Affymetrix Mouse Gene 1.0 ST whole genome arrays at 45°C for 16 h following procedures outlined in the GeneChip WT Terminal Labeling and Hybridization user manual. Arrays were post-processed on the Affymetrix GeneChip Fluidics Station 450, scanned on the GeneChip 3000 7G and the data extracted and processed using Affymetrix Command Console (version 3.1.1.1229) according to manufacturer's protocols. Resulting GeneChip.cel files were uploaded to iReport (Ingenuity Systems) or to Genesifter (Geospiza), and normalized via Robust Multi-array Average (RMA) using the default options of each system. Genes differentially expressed up or down >1.5-fold at *p* = 0.05) were identified using pairwise analysis (comparing WT to cMyBPC^−/−^ and/or comparing PND1 to PND9) or using two-way ANOVA (for multi-factoral analysis). *T*-test statistics were performed using a Benjamini and Hochberg correction.

### RT-qPCR

Whole hearts were dissected from E18.5, PND0, PND1, PND2, and PND9 pups generated from the breeding of heterozygous cMyBP-C^+/−^ adults, and snap-frozen in liquid nitrogen. RNA isolation and qRT-PCR were performed on hearts of each age and genotype according to standard methods, as described in Supplementary Methods.

### Immunohistochemistry for cell cycling marker on paraffin-embedded hearts

IHC on paraffin-embedded hearts were performed according to standard methods as detailed in Supplementary Methods, using the following antibodies: 1:20 anti-α-tropomyosin (CH-1; Developmental Studies Hybridoma Bank, University of Iowa, IA) to label myocardium, and 1:200 anti-Ki67 rabbit polyclonal antibody (Abcam, ab15580) to label cells actively participating in the cell cycle (**Figure 2**). For secondary detection, sections were incubated in 1:200 AlexaFluor 568 goat anti-mouse IgG_1_ and Alexafluor 488 goat anti-rabbit IgG_(*H*+*L*)_secondary antibodies (Molecular Probes, Eugene, OR, USA). In each case, as a control, similar sections were incubated with secondary antibodies only. Sections were coverslipped using warmed ProLong Gold Antifade Reagent (Invitrogen) with 4′,6-diamidino-2-phenylindole (DAPI) to label nuclei.

### Analysis of cell cycling by IHC

The number of cycling cardiomyocytes was determined in images of LV and septum from coronal sections labeled with antibodies against α-tropomyosin and the Ki-67 antigen, and counterstained with DAPI. Ki-67- and α-tropomyosin-labeled cells were counted in representative sections of at least three hearts of each genotype and age, and the mean calculated. Cells labeled with Ki-67 but not α-tropomyosin were designated as non-cardiomyocytes and thus excluded from counts. All calculations were subjected to statistical analysis using standard Students' *t*-test.

### Cryogenic preservation of hearts, cryosectioning, and IHC

Hearts were cryogenically preserved and cryosectioned according to standard procedures detailed in Supplementary Methods. Sections were mounted on glass slides and IHC was performed using 1:1,000 anti-α-actinin mouse monocolonal antibody (Sigma, A7811) to label cardiomyocytes, followed by AlexaFluor 568 goat anti-mouse IgG_1_ (Molecular Probes) at 1:250. Following IHC, sections were incubated for 10 min with 5 μg/ml wheat germ agglutinin (WGA)—Alexa 647 [Molecular Probes; for the determination of cross-sectional area (CSA)] in 1X PBS.

### Measurements of hypertrophy in cryogenic heart sections

For analysis of cardiomyocyte hypertrophy from cryogenic heart sections, measurements were taken from three hearts per genotype/age, with sampling from three cryosections per heart and three fields per section. The investigator performing the measurements and counts was blind to the genotype of all heart sections.

Hypertrophy was determined by measuring cardiomyocyte CSA on images of transversely cut heart cryosections labeled with anti-α-actinin, WGA-Alexa 647, and DAPI. Only images from LV and septum were imaged and quantified. Cells used in the analysis were confirmed as cardiomyocytes by the presence of α-actinin labeling. Manual tracing of the cardiomyocyte cross-sections was performed using the NIS-Elements 4.0 software which then calculated the CSA of individual cardiomyocytes. Ninety cardiomyocyte CSAs were measured per genotype/age. CSAs were averaged for each individual heart, and means were compared between genotypes within each age. All calculations were analyzed using Students' *t*-test.

### Measurements of hypertrophy in fixed, dissociated cardiomyocytes

In a method adapted from Mollova et al. (2013) with some modifications, hearts freshly isolated from PND2 and PND9 mice were rinsed in 1X PBS with 30 mM 2,3-butanedione monoxime (BDM, Sigma). Left ventricles (LV) + septums were isolated, minced and fixed in 2% formaldehyde for at least 45 min. After fixation, minced LV + septum were washed 3 × 5 min in 1X PBS, then incubated in 250 μl (PND2) or 500 μl (PND9) Type II collagenase (Gibco) at 37°C for 1–4 h for digestion. After digestion, cells were gently triturated with a plastic transfer pipet to disperse cells. Fetal bovine serum (FBS, Hyclone) was added to the cell suspension to reach a final concentration of 10% FBS to quench collagenase activity. Sodium azide (Spectrum) was added for a final concentration of 0.03% to inhibit bacterial growth. A drop of cell suspension was placed on a labeled glass slide and covered with a coverslip that was then sealed with nail polish. Cells were then imaged in bright field at 40X as described in Supplementary Methods.

### Data analyses

Values are reported as means ± SE. Statistical analyses were performed using SPSS (IBM) or SAS software version 9.3. For comparisons in which there were only two groups (WT vs. cMyBP-C^−/−^), Students' *t*-test was performed within each age group. For comparisons of three groups (WT, cMyBP-C^+/−^, cMyBP-C^−/−^), one-way ANOVAs were conducted with a *post-hoc* Bonferroni correction to compare between genotypes at each time point. For analysis of cell size on fixed, isolated cardiomyocytes, a compound symmetry correlation structure was used to account for repeated measures from one heart (*n* = 50–100 cells/heart) and to account for any litter effect. Significance is reported at *p* < 0.05. All graphs were generated using GraphPad Prism.

## Results

As described in the Methods Section, all comparisons were made using littermate offspring from heterozygous (cMyBP-C^+/−^) breedings to achieve more tightly controlled experimental conditions and reduce variance.

### cMyBPC-null hearts demonstrate a rapid increase in size and loss of function over first 10 days of life

**Figure 1:**
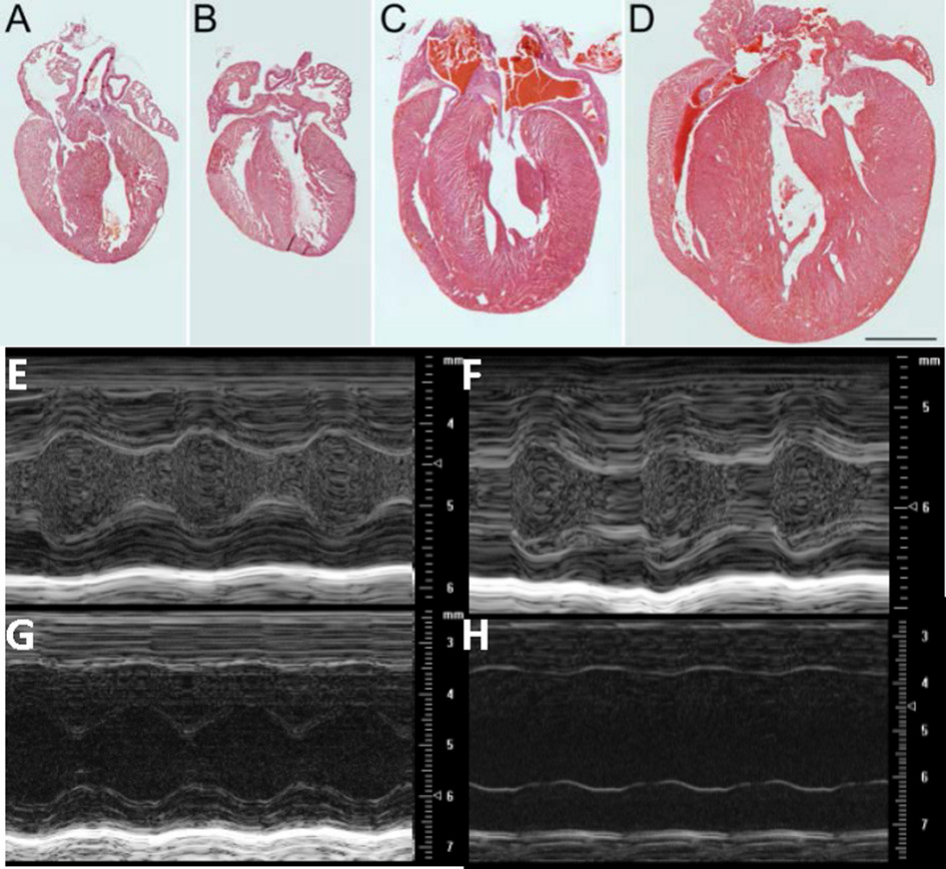
Morphologic and functional change in cMyBP-C^−/−^ between PND 1 and PND9: Hematoxylin and Eosin (H&E) staining of representative sections from hearts of WT **(A, C)** and cMyBP-C^−/−^ **(B, D)** mice at PND1 **(A,B)** and PND9 **(C,D)**. Scale bar = 1 mm. **(E–H)** Representative M-mode echocardiographic images of WT (*n* = 6; **E,G**) and cMyBP-C^−/−^ (*n* = 7; **F,H**) mice at PND1 **(E,F)** and PND9 **(G,H)**. Note different scales for PND1 vs. PND9. Measurements listed in Table 1. PND1 images show increased echogenicity within LV cavity due to nucleated red blood cells that are absent by PND9.

There was no evidence of gross morphological differences at PND1 (1 day after birth) between hearts from cMyBP-C^−/−^mice and those of WT littermates (Figures 1A,B). In sharp contrast, by PND9 there was a pronounced difference in the thickness of the interventricular septum and LVFWin cMyBP-C^−/−^ hearts compared to the hearts of WT littermates (Figures 1C,D). The HW to BW ratio was significantly elevated in PND9 cMyBP-C^−/−^ pups vs. WT [(%) 0.624 ± 0.015 vs. 0.501 ± 0.0062, respectively, ± SE, *p* < 0.001, *n* = 13 (cMyBP-C^−/−^), 32 (WT)] but was not different at PND1 [(%) 0.602 ± 0.007 vs. 0.619 ± 0.01, ± SE, *p* = 0.833, *n* = 18 (cMyBP-C^−/−^), 13 (WT)], which has been previously observed (de Lange et al., 2013). These morphologic changes at PND9 are consistent with the described hypertrophic phenotype of this mouse model (Harris et al., 2002; de Lange et al., 2013).

The gross morphology was supported by echocardiography, which revealed no difference at PND1 between WT and cMyBP-C^−/−^ hearts with regard to wall thickness and chamber diameter (Figures 1E–H and Table 1), except for a minimal but significantly lower left ventricular posterior wall thickness during systole (LVPW_*s*_) at PND1 in cMyBP-C^−/−^ vs. WT hearts (Table 1). This difference was not present during diastole or in the anterior wall. At a functional level, cMyBP-C^−/−^ mice showed no difference in ejection fraction at the same age. However, while ejection time appeared to be similar between WT and cMyBP-C^−/−^ mice at PND1, isovolumic relaxation time (IVRT) was longer in cMyBP-C^−/−^ pups, suggesting an early subtle functional deficit (Table 1) which was explored previously (de Lange et al., 2013).

**Table 1:** Echocardiographic Measurements in WT and cMyBP-C ^−/−^Mice at PND1 and PND9.

	**PND1**		**PND9**	
	**WT (*N* = 6)**	**cMyBP-C^−/−^ (*N* = 7)**	**WT (*N* = 6)**	**cMyBP-C^−/−^ (*N* = 7)**
LVPW; d (mm)	0.32 ± 0.03	0.29 ± 0.04	0.44 ± 0.07	0.71 ± 0.15^*^
LVPW; s (mm)	0.50 ± 0.04	0.44 ± 0.04^*^	0.67 ± 0.11	0.86 ± 0.17^*^
LVAW; d (mm)	0.30 ± 0.04	0.32 ± 0.02	0.48 ± 0.07	0.68 ± 0.13^*^
LVAW; s (mm)	0.47 ± 0.07	0.47 ± 0.05	0.69 ± 0.19	0.91 ± 0.11^*^
LVID; d (mm)	1.57 ± 0.23	1.45 ± 0.31	1.99 ± 0.51	2.98 ± 0.46^*^
LVID; s (mm)	0.94 ± 0.26	1.01 ± 0.31	1.19 ± 0.50	2.56 ± 0.48^*^
LV mass; (mg)	7.08 ± 2.11	6.28 ± 2.60	18.16 ± 7.01	60.65 ± 16.11^*^
Ejection Fraction (%)	73.86 ± 11.05	62.65 ± 11.90	74.4 ± 13.24	32.13 ± 9.59^*^
IVRT (ms)	26.46 ± 3.48	39.68 ± 5.36^*^	20.17 ± 3.48	37.96 ± 2.99^*^
Ejection Time (ms)	55.13 ± 6.14	52.69 ± 11.66	44.89 ± 6.13	30.97 ± 3.64^*^
Heart Rate (bpm)	450 ± 27	452 ± 59	502 ± 38	473 ± 35

*Values are means ±SD. LVPW, left ventricular posterior wall thickness; d, diastole; mm, millimeters; s, systole; LVAW, left ventricular anterior wall thickness; LVID, left ventricular interior diameter; IVS, intraventricular septum; mg, milligrams; IVRT, isovolumetric relaxation time; ms, milliseconds; bpm, beats per minute. n = 6 (WT), n = 7 (cMyBP-C^−/−^)*.

* *p < 0.05 vs. age-matched WT. PND9, but not PND1, mice were anesthetized with isoflurane*.

In sharp contrast to the virtual absence of morphological differences at PND1, by PND9 the cMyBP-C^−/−^ ventricle walls were considerably thicker than WT (Figures 1E–H and Table 1), consistent with the gross morphology. Measured during systole, the cMyBP-C^−/−^ anterior and posterior left ventricular wall thicknesses (LVAW and LVPW) were increased 32% and 29%, respectively, compared to WT hearts. The left ventricular internal diameter during diastole (LVID_d_) and systole (LVID_s_) was also significantly larger in the cMyBP-C^−/−^ mice at PND9 vs. WT, suggesting that dilation exists at this early stage of the disease. Furthermore, a functional deficit virtually absent at PND1 was readily apparent in the PND9 cMyBP-C^−/−^ mice. The ejection fraction was considerably lower (43% of WT), and the isovolumic relaxation time significantly prolonged (188% of WT), with a shortened ejection time (69% of WT) in the PND9 cMyBP-C^−/−^ hearts (Table 1).

### At PND1, microarray analysis of cMyBPC^−/−^ mice reveals upregulation of genes involved in cell cycling and proliferation

Pairwise comparison using analysis software (iReport and Genesifter) of microarray data at PND1 identified many genes in the cell cycle pathway that were upregulated >1.5-fold (*p* < 0.05) in the cMyBP-C^−/−^ hearts vs. (vs.) WT, indicating either an increase of cell cycling or a delay/persistence of developmental processes, or a combination of both. Genesifter analysis revealed that the highest scoring pathway in KEGG (*The Kyoto Encyclopedia of Genes and Genomes*) was the “cell cycle” pathway, which includes the differential expression of genes involved in several pathways linked to cell division, replication, and proliferation (Table 2).

**Table 2:** “Cell Cycle” is the Most Differentially Regulated Pathway Between cMyBP-C^−/−^ and WT Hearts at PND1.

**KEGG Pathway**	**Gene name**	**Gene ID**	**Direction**	Ratio^*^	***p*-value**
Cell Cycle	Cyclin B1	Ccnb1	Up	1.88	0.006
	Cell division cycle 25 homolog C (*S. pombe*)	Cdc25c	Up	1.72	0.016
	Dynactin 5, mRNA	Plk1	Up	1.69	0.004
	Cyclin B2	Ccnb2	Up	1.63	0.003
	Cyclin A2	Ccna2	Up	1.58	0.001
	Cell cycle protein P55CDC	Cdc20	Up	1.57	0.023
	Cell division cycle 2 homolog A (*S. pombe*)	Cdc2a	Up	1.55	0.010
	Pituitary tumor-transforming gene1	Pttg1	Up	1.54	0.005
	Budding uninhibited by benzimidazoles 1 homolog, beta (*S. cerevisiae*)	Bub1b	Up	1.52	0.011
	Budding uninhibited by benzimidazoles 1 homolog, beta (*S. cerevisiae*)	Bub1	Up	1.50	0.037

* *Ratio represents the gene expression of cMyBP-C^−/−^ divided by gene expression of WT. Thus, a ratio of 1.88, as in Cyclin B1, is equivalent to an 88% increase in RNA expression in the cMyBP-C^−/−^ hearts vs. WT*.

Data from pairwise analysis of PND1, WT vs. cMyBP-C^−/−^ hearts were further analyzed using Ingenuity Pathway Analysis (IPA, Ingenuity Systems). This analysis identified additional cell cycle pathway genes showing differential regulation, including but not limited to cyclin dependent kinase (*Cdk*), the anaphase-promoting complex (*APC*), importin α and β, and the extracellular-signal-regulated kinases (*Erk1/2*; Supplementary Figure 1, Supplementary Table 1). Taken together, the significant upregulation of genes involved in cell cycle, replication, and proliferation in cMyBP-C^−/−^ mice compared to WT at PND1 suggests an early postnatal increase in cardiomyocyte cell cycling that precedes the hypertrophic phenotype.

### Microarray analysis reveals a switch to hypertrophic signaling in cMyBP-C^−/−^ vs. WT by PND9

Pairwise analysis of microarray data comparing WT and cMyBP-C^−/−^ hearts at PND9 revealed that the KEGG “cell cycle” pathway was no longer upregulated in cMyBP-C^−/−^ hearts. In addition, none of the genes implicated in cell division, replication, and proliferation that were identified at PND1 were differentially expressed at PND9. In the PND9 hearts, the top scoring KEGG pathway was “hypertrophic cardiomyopathy,” which included eight upregulated genes and 1 (other than cMyBP-C itself) that was downregulated >1.5-fold at *p* = 0.05 (Supplementary Table 2). Supplementary Table 3 lists the most differentially regulated KEGG pathways in addition to “hypertrophic cardiomyopathy,” between WT and cMyBP-C^−/−^ mice at PND9, along with their corresponding genes, as identified by Genesifter analysis. Genes listed in pathways outside of “hypertrophic cardiomyopathy” are nonetheless directly implicated in HCM or other cardiovascular diseases (Weizmann Institute of Science MalaCards Human Malady Compendium and/or the University of Copenhagen DISEASES database). Hypertrophic pathways were neither activated nor differentially regulated between WT and cMyBP-C^−/−^ mice at PND1.

### Cardiomyocyte cell cycling is increased in cardiomyocytes from PND1 cMyBP-C^−/−^ mice

**Figure 2:**
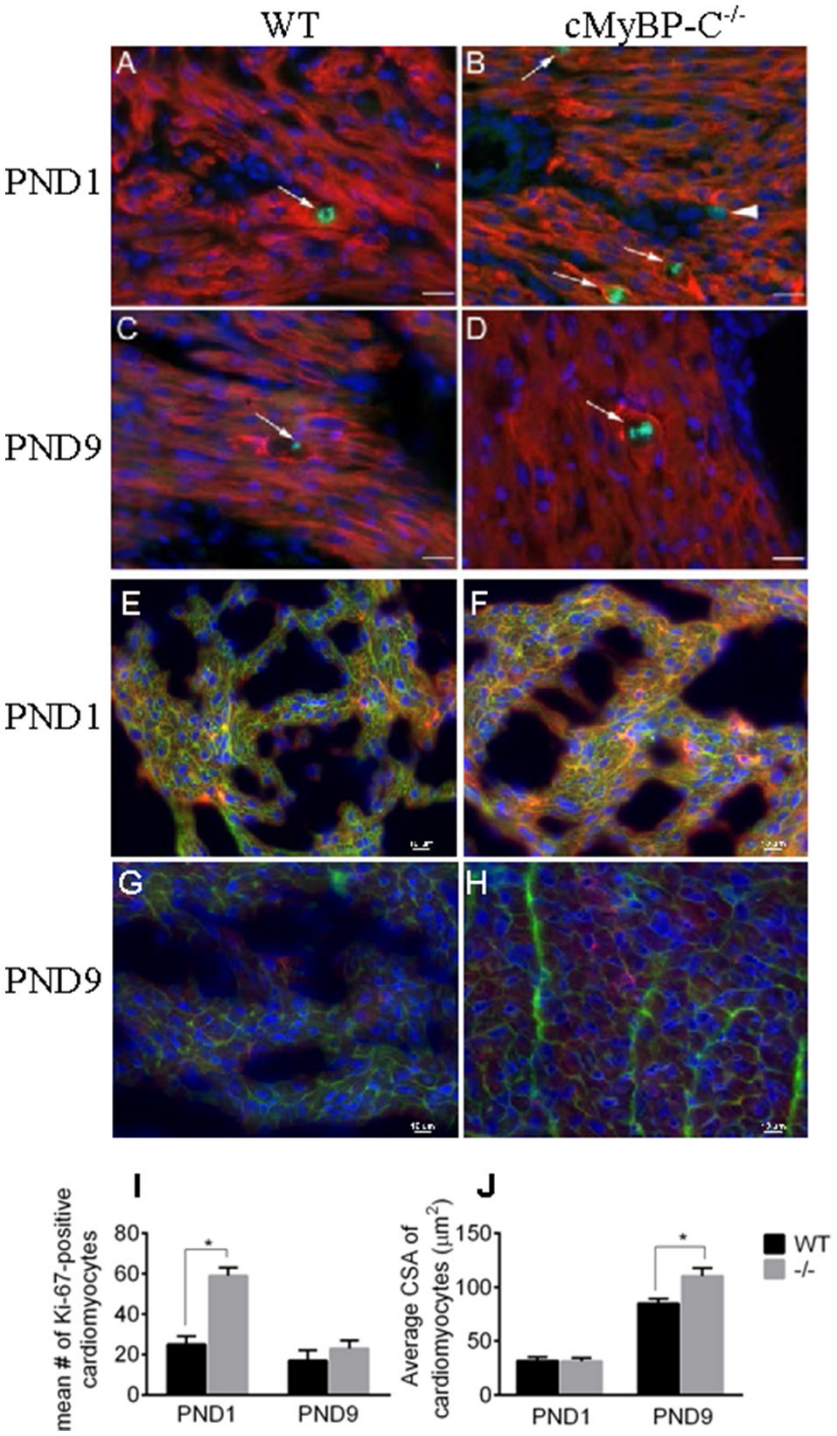
Hyperplasia precedes hypertrophy in cMyBP-C^−/−^ hearts. **(A–D)** Representative sections of hearts of WT **(A,C)** and cMyBP-C^−/−^ **(B,D)** mice at PND1 **(A,B)** and PND9 **(C,D)** labeled with α-tropomyosin (red), a muscle-specific marker, Ki-67 (green) to highlight active cell cycling, and counterstained with DAPI (blue) to highlight nuclei. Arrows highlight single Ki-67-expressing cardiomyocytes in each panel. Arrowhead in **(B)** highlights a Ki-67-positive, α-tropomyosin-negative, non-cardiomyocyte cell that was excluded from counts. Scale bars = 20 μm. **E–H**: Representative sections of WT **(E,G)** and cMyBP-C^−/−^ **(F,H)** mouse hearts at PND1 **(E,F)** and PND9 **(G,H)** labeled with α-actinin (red) and WGA AlexaFluor 647 (pink) to highlight cell borders, and counterstained with DAPI (blue). Quantification of number of Ki-67-positive cardiomyocytes **(I)** and cardiomyocyte cross-sectional area (CSA; **J**) for WT and cMyBP-C^−/−^ (-/-) at PND1 and PND9 in representative heart sections, as shown in **(A-H)**. Means ±SE are reported, ^*^p < 0.05.

To confirm the upregulation of cell cycling noted at the RNA level in the microarray in PND1 cMyBP-C^−/−^ mice, we performed IHC on heart sections from WT and cMyBP-C^−/−^ mice. Coronal, paraffin-embedded heart sections were labeled with an anti-α-tropomyosin antibody, identifying cardiac, and skeletal muscle cells. Co-labeling using the Ki-67 antibody (Figures 2A–D) revealed a significantly higher number of cardiomyocytes actively participating in the cell cycle in the PND1 cMyBP-C^−/−^ hearts compared to that of WT littermates (59 ±4 vs. 25 ±4 Ki67-positive cardiomyocytes per section, *n* = 3 hearts per group, ±SE, *p* < 0.05, Figure 2I), in agreement with the microarray data. At PND9, further corroborating the microarray data, there was no statistical difference in the number of Ki67-positive cardiomyocytes in cMyBP-C^−/−^ vs. WT hearts (23 ±4 vs. 17 ±5 cells per section, ± SE, *p* = 0.38, *n* = 3 hearts per group, Figure 2I). These data support elevated cell cycling of early postnatal cardiomyocytes in cMyBP-C^−/−^ hearts that is absent by PND9.

### Cardiomyocyte hypertrophy evident in PND9 but not PND1 cMyBP-C^−/−^ hearts

To quantify the degree of hypertrophy of the cardiomyocytes at both PND1 and PND9, we determined cardiomyocyte size using cryopreserved WT and cMyBP-C^−/−^ hearts. Hearts were cut in transverse sections and immunolabeled with WGA attached to the fluorophore Alexa 647 to visualize cell borders, anti-α-actinin to confirm cardiomyocyte cell type, and DAPI to visualize nuclei (Figures 2E–H). We measured cardiomyocyte cross-sectional area in 90 α-actinin-positive cells per heart from multiple individual sections from WT (*n* = 3 hearts) and cMyBP-C^−/−^ littermates (*n* = 3 hearts). Averaged cross-sectional areas of individual myocytes are depicted in Figure 2J. There was no indication of hypertrophy at PND1 in cMyBP-C^−/−^ hearts, evidenced by similar cardiomyocyte mean cross-sectional area vs. WT (31.30 ± 3.19 vs. 31.76 ± 3.60 μm^2^, respectively, ±SE, *p* = 0.93). In contrast, at PND9 cardiomyocytes from cMyBP-C^−/−^ hearts were significantly hypertrophied compared to those from WT hearts, with a 30% higher mean cardiomyocyte cross-sectional area (110.30 ± 7.50 vs. 85.04 ± 4.35 μm^2^, ±SE, *p* < 0.05, Figure 2J).

Since cardiac fibrosis is often found in HCM and might contribute to heart mass and size, we stained sections of PND1 and PND9 hearts using Masson's Trichrome and Elastic Trichrome (Supplementary Figure 2). No difference in interstitial fibrosis or disarray was observed between genotypes at either age.

### Timeline of cell cycling and onset of hypertrophy

The microarray and IHC data revealed that elevated cell cycling preceded the hypertrophic response, and that the enhanced cell cycling had normalized by PND9. These data raised the question of if differential hyperplasia precedes birth, and also how long it persists after PND1. The time course duration for cell cycling could have profound implications on the final cardiomyocyte number present in the cMyBP-C^−/−^ hearts. Furthermore, the temporal relationship of potential hyperplastic vs. hypertrophic cellular signaling could lend insight as to a potential interaction between these two processes. Therefore, we expanded our study to include the investigation of cardiac morphology and gene expression at the added time points of embryonic day 18.5 (E18.5), PND0, and PND2 as well as at the previously established time points of PND1 and PND9, on an additional set of animals. Furthermore, we investigated heterozygote (cMyBP-C^+/−^) mice to determine whether any early, mild changes were present in these animals.

### Timeline of gross hypertrophy

**Figure 3:**
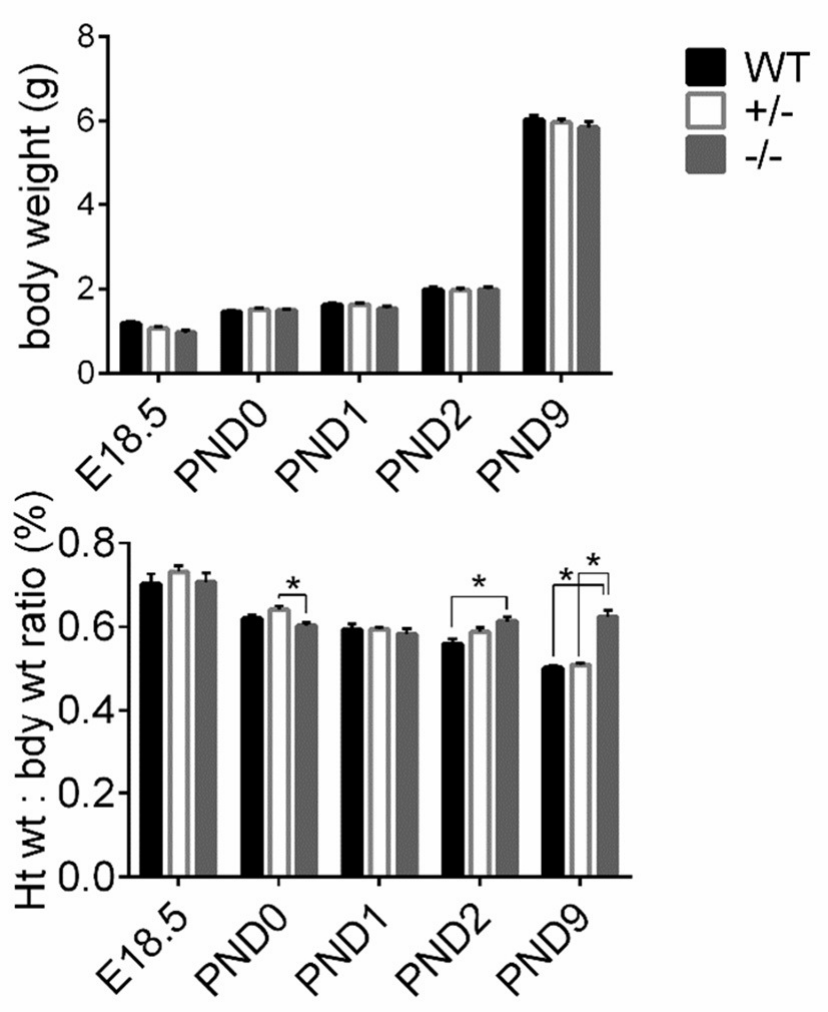
Assessment of gross cardiac hypertrophy in WT, cMyBP-C^+/−^ (+/^−^), and cMyBP-C^−/−^ (-/-) hearts from E18.5 to PND9. (Top) body weight and (bottom) heart weight to body weight ratio. Numbers of pups/hearts per day/genotype are listed in Supplementary Table 4. Means ±SE are reported, ^*^p < 0.05.

To assess gross cardiac hypertrophy, animals and hearts were weighed at their respective time points and HW to BW ratios were calculated, as previously. There were no differences in pup weights among WT, cMyBP-C^+/−^ or cMyBP-C^−/−^ mice at any time point, suggesting that the cMyBP-C^−/−^ mice are not globally growth retarded (Figure 3, top). HW to BW ratios of cMyBP-C^+/−^ mice did not differ from WT at any time point. cMyBP-C^−/−^ HW to BW ratios were not different than WT at E18.5, PND0, or PND1. However, cMyBP-C^−/−^ pups had higher HW to BW ratios than their time-matched WT littermates at the remaining time points of PND2 (%, 0.61 ±0.01 vs. 0.57 ±0.01, ±SE, respectively) and PND9 (%, 0.64 ±0.02 vs. 0.51 ±0.01, respectively, ±SE, *P* < 0.001; Figure 3, bottom), suggesting that gross cardiac hypertrophy exists at the organ level starting at PND2 in the homozygote null animals (See Supplementary Table 4 for numbers of mice per genotype/day point).

### Timeline of cellular hypertrophy using genetic markers

**Figure 4:**
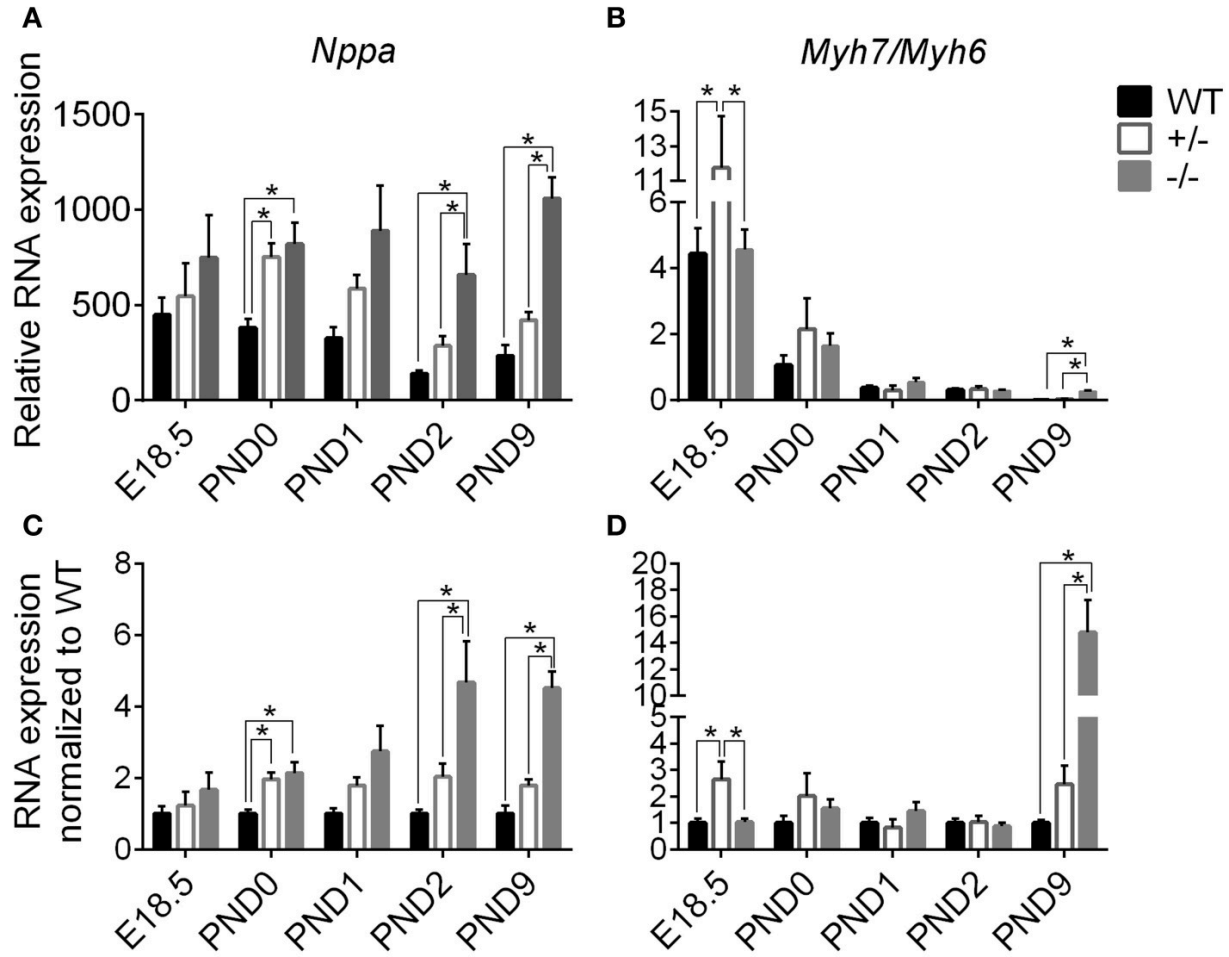
Markers of stress and hypertrophy in WT, cMyBP-C^+/−^ (+/^−^), and cMyBP-C^−/−^ (-/-) from E18.5 to PND9. RNA expression of *Nppa* **(A,C)** and the ratio of *Myh7/Myh6* RNA expression **(B,D)** relative to the average of 2 housekeeping genes, *Gapdh* and ß*-actin* **(A,B)** and normalized to WT hearts at each corresponding time point **(C,D)**. Inset in B is an expanded view of PND9. *n* = 5 hearts/group. Means ±SE are reported, ^*^p < 0.05.

Since gross cardiac enlargement may or may not be the result of cellular hypertrophy, we evaluated the timing of the onset of cardiomyoctye hypertrophy by assessing expression of two genes widely recognized as hypertrophic markers, *Nppa* (atrial natriuretic peptide) and *Myh7* (expressed as the ratio of *Myh7* [β-myosin heavy chain]*/Myh6* [α-myosin heavy chain]), at the added time points starting before birth. Figure 4 shows expression of *Nppa* (A) and *Myh7/Myh6* (B) relative to the housekeeping genes *Gapdh* and ß*-actin*, as well as when normalized to WT levels (C, D), measured through targeted qPCR (Separated *Myh6* and *Myh7* expression is shown in Supplementary Figure 3). Expression of *Nppa* was higher in cMyBP-C^−/−^ and cMyBP-C^+/−^ vs. WT hearts at PND0, prior to the onset of gross cardiac hypertrophy in cMyBP-C^−/−^ mice (see Figure 3, bottom), and was also higher at PND2 and PND9 in cMyBP-C^−/−^ hearts (Figures 4A,C). Although there were trends toward elevated *Nppa* both prior to birth (E18.5) and at PND1 in cMyBP-C^−/−^ hearts, they were not statistically significant. The early elevation of *Nppa*, prior to gross hypertrophy in the cMyBP-C^−/−^ mouse, and in the absence of gross hypertrophy in the cMyBP-C^+/−^ mouse, raises the question of whether *Nppa* is a true marker for hypertrophy or rather a non-specific response to stress.

cMyBP-C^+/−^ hearts showed elevated *Myh7/Myh6* expression at E18.5 but at no other time point. Expression of *Myh7/Myh6* in cMyBP-C^−/−^ hearts was only elevated at PND9 (Figures 4B,D), although HW to BW ratio was increased at PND2 (see Figure 3, bottom). The lack of an elevated *Myh7/Myh6* ratio at PND2 suggests that the increase in HW to BW ratio at PND2 may not be due to cellular hypertrophy but could be the result of cardiomyocyte hyperplasia, resulting from the observed increase in cell cycling.

### Timeline of cardiomyocyte cell cycling

**Figure 5:**
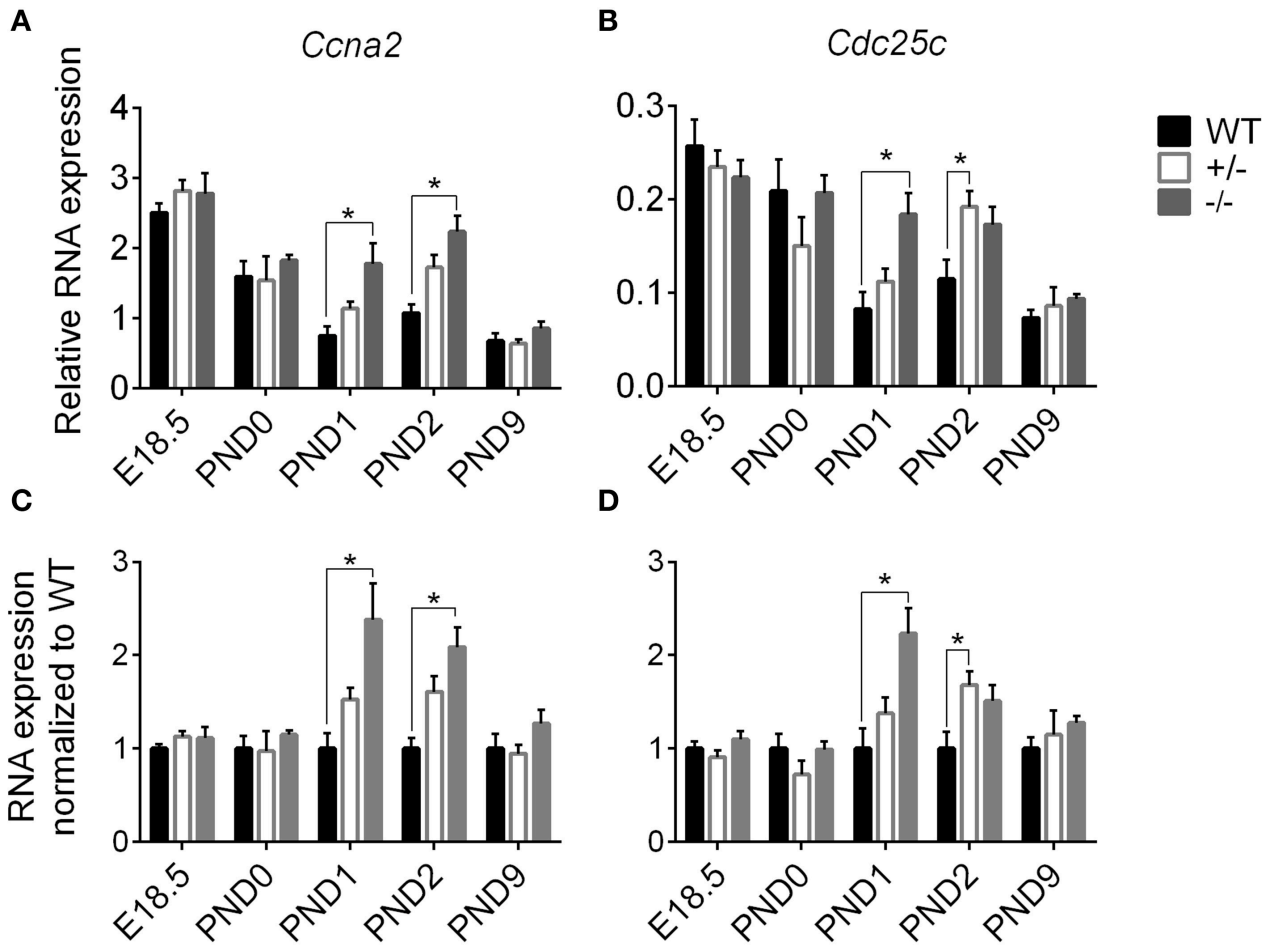
Higher cell cycling gene expression in cMyBP-C^−/−^ vs. WT hearts. RNA expression of *Ccna2* **(A,C)** and *Cdc25c* **(B,D)** relative to the average of 2 housekeeping genes, *Gapdh* and ß*-actin* **(A,B)** and normalized to WT hearts **(C,D)**. *n* = 5 hearts/group. Means ±SE are reported, ^*^p < 0.05.

We performed targeted qPCR at the expanded time points starting before birth to determine whether the enhanced postnatal cardiomyocyte cell cycling in cMyBP-C^−/−^ hearts is a prolongation of the highly proliferative embryonic state or represents an independent postnatal increase. RNA expression was quantified for two cell cycling genes, cyclin A2 (*Ccna2*) and cell division cycle 25c (*Cdc25c*), both of which showed higher levels of expression in cMyBP-C^−/−^ hearts at PND1 (Table 2) but no difference at PND9 in the microarray analysis. Targeted qPCR revealed that RNA expression levels of *Ccna2* and *Cdc25c* were not different at E18.5 or PND0 between cMyBP-C^−/−^ and WT hearts (Figure 5). However, at PND1, levels of expression of *Ccna2* and *Cdc25c* were maintained at a higher level in cMyBP-C^−/−^ hearts vs. WT (elevated 2.38 ± 0.39- and 2.23 ± 0.28-fold vs. WT, respectively). This elevated RNA expression continued through PND2 for *Ccna2* (Figures 5A,C). Although the gene expression of *Cdc25c* trended toward being higher in cMyBP-C^−/−^ vs. WT hearts at PND2, the difference was not significant at this time point. This elevated cell cycling in cMyBP-C^−/−^ vs. WT hearts was also supported by increased IHC detection of Ki-67 in cMyBP-C^−/−^ mice (Supplementary Figures 4A,B,E).

The cMyBP-C^+/−^ hearts showed no difference in *Ccna2* expression at any time point, and showed elevated *Cdc25c* expression compared to WT only at PND2. However, there was a trend toward upregulation for *Cdc25c* at PND1 and PND2, and for *Ccna2* at PND1. At PND9, RNA expression in the cMyBP-C^−/−^ and cMyBP-C^+/−^ hearts was not different from WT for either of the cell cycling genes. Thus, elevated expression of cell cycling genes in cMyBP-C^−/−^ hearts appears to reflect a prolongation of physiologic neonatal cell cycling, while cMyBP-C^+/−^ hearts show a trend, though mostly non-significant, in the same direction as cMyBP-C^−/−^ hearts.

Increased cell cycling in the cMyBP-C^−/−^ cardiomyocytes does not necessarily indicate increased cardiomyocyte proliferation. However, unless cellular hypertrophy is present at PND2, the increased HW to BW ratio at PND2 would only be reasonably explained by increased cardiomyocyte proliferation. The absence of an elevated *Myh7/Myh6* expression at PND2 raises doubt as to if cellular hypertrophy exists at PND2 in the cMyBP-C^−/−^ heart.

### Assessment of cardiomyocyte size at PND2 and PND9

**Figure 6:**
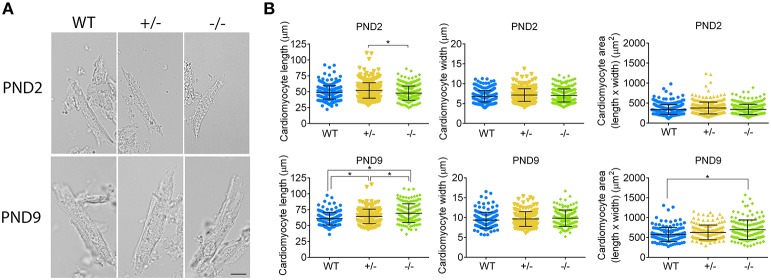
Cardiomyocyte hypertrophy is present at PND9 but not PND2 in cMyBP-C^−/−^ hearts. **(A)** Representative cardiomyocytes from WT, cMyBP-C^+/−^ (+/^−^), and cMyBP-C^−/−^ (-/-) mice at PND2 and PND9. Scale bars = 10 μm. **(B)** Cardiomyocyte length, width, and area (length × width) plotted for each individual cell with horizontal line at the mean ±SD. *n* = 100 cells/heart (PND2), *n* = 50 cells/heart (PND9), *n* = 3 hearts/genotype. ^*^p < 0.05.

To confirm whether or not cardiomyocyte hypertrophy is present in the PND2 cMyBP-C^−/−^ LV to contribute to the increased HW to BW ratio, we directly assessed cardiomyocyte size using a method adapted with modifications from Mollova et al. (2013). Freshly excised hearts were fixed *in situ* with formaldehyde prior to collagenase digestion, to yield striated, rod-shaped cardiomyocytes (Figure 6A). Cells were then imaged and measured for length and width. Cardiomyocyte area was calculated as the product of length × width. At PND2, cMyBP-C^+/−^ cardiomyocytes were slightly longer in length compared to the cMyBP-C^−/−^ cardiomyocytes, but were not different from WT (Figure 6B and Supplementary Table 5). Cardiomyocytes from cMyBP-C^−/−^ LVs were not different from WT cardiomyocytes in length, width, or area, demonstrating that cardiomyocyte hypertrophy is not responsible for the increased HW to BW ratio in cMyBP-C^−/−^ mice at PND2. The lack of cardiomyocyte hypertrophy at PND2 was further supported by measurements of cardiomyocyte CSA in IHC (Supplementary Figures 4C,D,F).

At PND9, cardiomyocytes from cMyBP-C^−/−^ LVs were longer than WT and cMyBP-C^+/−^ cardiomyocytes, leading to an increased cell area compared to WT (Figure 6B, Supplementary Table 5). No changes in cell width were observed. Thus, at PND9, cardiomyocyte hypertrophy is at least partly responsible for the increased HW to BW ratio in cMyBP-C^−/−^ hearts, as expected.

These data indirectly suggest that the increased cell cycling observed by microarray (Table 2, Supplementary Figure 1) RNA expression (Figure 5), and IHC (Figure 2, Supplementary Figures 4A,B,E) does in fact lead to cardiomyocyte proliferation, as further suggested by increased expression of genes involved in the cytokinetic processes of contractile ring assembly or in abcission (Supplementary Table 6). This proliferative response is substantial enough to be wholly responsible for the increased HW to BW ratio observed at PND2 in the cMyBP-C^−/−^ cardiomyocytes, as cardiomyocyte hypertrophy is not present at PND2. By PND9, cardiomyocyte hypertrophy is present in the cMyBP-C^−/−^ but not cMyBP-C^+/−^ LVs, as indicated by cardiomyocyte area (Figure 6).

## Discussion

We conclude from the data presented here that in an HCM model in which mice lack cMyBP-C there is a prolongation of postnatal cell cycling which results in increased cardiomyocyte proliferation, prior to activation of the hypertrophic response. These data further reveal that cellular signaling in the heterozygote (cMyBP-C^+/−^) heart trends in the same direction as the cMyBP-C^−/−^ heart, but fails to elicit a hypertrophic phenotype by PND9. At 6 months of age, however, the cMyBP-C^+/−^ mice show evidence of diastolic dysfunction, altered contractile kinetics, and pro-arrhythmic changes in their action potential, while remaining free of overt hypertrophy (Cheng et al., 2013). It is unknown whether the cMyBP-C^+/−^ mice develop hypertrophy at a more advanced age.

We previously reported that newborn cMyBP-C^−/−^ mice are grossly indistinguishable from their WT littermates, but by PND9 the cMyBP-C^−/−^ hearts are significantly enlarged (de Lange et al., 2013). This response has been assumed to be purely hypertrophic. In performing a transcriptome comparison of newborn and PND9 mice from cMyBP-C^−/−^ and WT pups, we expected to identify early hypertrophic signaling events. Unexpectedly, the data presented here, while confirming that germline ablation of cMyBP-C^−/−^ does not cause any apparent overt pathology during cardiogenesis, does however indicate a prolonged phase of increased cell cycling and proliferation postnatally that precedes the significant cellular hypertrophic response apparent by PND9. While we recognize that counting cardiomyocyte number is the most direct method to assess proliferation, it is technically unfeasible in our experimental design. The pups in this study are obtained from heterozygote × heterozygote breedings, such that all litter mates are processed in parallel, with genotypes unknown at the time of harvest. Additionally, recovery and counting of cardiomyocytes from PND2 hearts proved highly unreliable and irreproducible. Therefore, we relied upon several indirect measures to differentiate a proliferative vs. hypertrophic response, including increased HW to BW ratio in the PND2 cMyBP-C^−/−^ pups (*n* = 25) vs. WT (*n* = 24) in the absence of cardiomyocyte hypertrophy, increased cardiomyocyte Ki-67 positivity, and direct measure of cardiomyocyte areas.

### The potential contribution of cardiomyocyte proliferation to heart size

Under normal processes of mammalian development, the fetal heart increases its size primarily through cell division, but transitions shortly after birth to physiologic hypertrophic growth to correlate heart size with somatic growth (Walsh et al., 2010; Porrello et al., 2011). In the context of HCM, elevated and persistent proliferation may have a significant impact on eventual disease severity. Enhanced early cardiomyocyte cell cycling at PND1 and PND2 in the cMyBP-C^−/−^ hearts that is significant enough to lead to an increase in HW to BW ratio in the absence of cardiomyocyte hypertrophy at PND2, as observed here, could provide an additional pool of myocytes that later participate in the hypertrophic response. The impact of prolonged cell division on total cardiomyocyte number may be substantial. Between PND1 and PND4 in normal hearts of WT mice, cardiomyocyte population increases by as much as 40% (Naqvi et al., 2014). Any additional increase and/or prolongation of this exponential proliferative capacity in the neonate would be expected to profoundly influence total cardiomyocyte number. Whether or not the early proliferative responses observed in our murine model occur in human myocardium to exert an as yet unappreciated influence in disease manifestation remains to be determined. However, there are data suggesting that the presence and importance of cardiomyocyte proliferation early in life extends to humans, and that cardiomyocyte proliferation before 20 years of age contributes to heart size (Mollova et al., 2013). The question of if the degree of proliferation is altered in HCM in humans remains unanswered.

### The perinatal period may provide a unique opportunity for modifying disease course

Evidence derived from other models of HCM further support the potential importance of this early pre-hypertrophic period in determining the course toward overt HCM (Jiang et al., 2013; Cannon et al., 2015). Silencing an HCM-causing Arg403Gln *Myh6* mutation prior to hypertrophy prevented the development of hypertrophy, while silencing the mutation after hypertrophy was already apparent did not lead to a reversal of the phenotype (Jiang et al., 2013). When the same mutation is suppressed from conception through 6 weeks of age, the degree of hypertrophy and fibrosis at 40 weeks was significantly attenuated compared to non-suppressed controls. When suppression occurred after 6 weeks of age, the mice developed severe hypertrophy (Cannon et al., 2015). Additionally, cardiac-specific ablation of cMyBP-C during adulthood results in significantly less cardiac hypertrophy compared to mice with germline ablation of cMyBP-C (Chen et al., 2012). These data suggest that the proliferative capacity of the neonatal cardiomyocyte provides a unique, potentially modifiable mechanism that contributes to HCM in a manner that is unparalleled later in life.

### Cell proliferation and cell hypertrophy: distinct or interrelated pathways?

The transition that we observe in the cMyBP-C^−/−^ hearts from an early elevation in cardiomyocyte proliferation, associated with an increased HW to BW ratio, to a later cardiomyocyte hypertrophy raises the question of whether the proliferative and hypertrophic pathways are distinct from each other or interrelated. The phenomenon of increased cardiomyocyte proliferation prior to cellular hypertrophy was recently reported to also occur in non-sarcomere etiologies of pathologic cardiac hypertrophy (Kim et al., 2014; Schipke et al., 2015). Enhanced cardiomyocyte proliferation in the first 2 weeks of life was found in both *Prkag2* cardiomyopathy, in which an adenosine monophosphate (AMP)-activated protein kinase mutation causes abnormal glycogen storage (Kim et al., 2014), as well as in mice with a deletion of guanylyl cyclase A (GC-A) that proceed to develop cardiac hypertrophy (Schipke et al., 2015). In both of these models, mice exhibited a prolonged phase of postnatal cardiomyocyte proliferation that occurred prior to the ensuing cellular hypertrophy, but coincident with an increased HW to BW ratio, echoing the results we obtained with the cMyBP-C^−/−^ mice. The authors concluded, as do we, that the postnatal elevation in proliferation contributed to the hypertrophic phenotype. The study presented here extends the observation of enhanced hyperplasia preceding cardiomyocyte hypertrophy to the disease of HCM and proposes that communication between the two mechanisms facilitates HCM progression. Together, these studies support a more generalizable role for a cardiac hyperplastic response to gene mutations associated with the later development of hypertrophy. These data imply that variations in the increased pool of cells available to participate in the hypertrophic process may influence the eventual severity of the phenotype, a process further modified by the specific HCM mutation and/or the genetic background of the individual.

While the relevance of an interaction between hyperplastic and hypertrophic cellular response pathways remains to be fully explored, the implication that underlying pathologic conditions can influence the timing of exit of cardiomyocytes from the cell cycle is intriguing. In chondroblasts, the hypertrophic phase of growth is unable to initiate until completion of the proliferative phase, suggesting either cross-talk between the pathways or an absolute requirement for exit from the cell cycle before hypertrophy can proceed (Omelyanenko et al., 2013). The overlap between many of the proteins involved in both of these processes implies additional nuance in their regulation.

### Heterogeneity in HCM

Recently, Jiang et al. (2015) reported the early postnatal phenotype of mice harboring a mutation that prematurely truncates cMyBP-C at amino acid 1,064 of 1,270 (cMyBP-C^t/t^). Although both the cMyBP-C^−/−^ and cMyBP-C^t/t^ exhibit an early postnatal hyperplastic response, resulting in a HW to BW ratio increase independent of cellular hypertrophy, the two mouse models also show some important divergent findings. While the cMyBP-C^−/−^ mouse in our study showed cardiomyocyte hypertrophy following the early elevated proliferation, the cMyBP-C^t/t^ mouse exhibited a purely hyperplastic response without any subsequent cardiomyocyte hypertrophy. The resulting phenotype is a dilated cardiomyopathy, as opposed to HCM. Furthermore, Jiang et al. suggest that in contrast to the homozygote cMyBP-C^t/t^ mouse, the hearts of individual human patients heterozygous for cMyBP-C mutations are purely hypertrophic, and the difference in allelic expression between the cMyBP-C^t/t^ and heterozygous patients results in divergent cellular pathways both leading to cardiomyopathy (Jiang et al., 2015). In contrast, mice in our study heterozygous for the null mutation (cMyBP-C^+/−^) appear to have an early intermediate expression between WT and cMyBP-C^−/−^ for genes involved in cell cycling and stress response, which then return to normal by PND9. These heterozygous mice ultimately fail to show a cardiomyopathic phenotype by PND9, but the trend in gene expression in the same direction as the cMyBP-C^−/−^ mice suggests similar, not divergent cellular signaling between cMyBP-C^−/−^ and cMyBP-C^+/−^. We propose that the difference in the cMyBP-C^−/−^ vs. cMyBP-C^t/t^ phenotypes may be a product of differences in expressed protein. Although the cMyBP-C^t/t^ mouse has been regarded as equivalent to a cMyBP-C null animal (Sadayappan et al., 2006; Tanner et al., 2014; Jiang et al., 2015), initial reports of this model show expressed protein in myofibrillar preparations, albeit at low levels (McConnell et al., 1999). We hypothesize that small amounts of expressed, truncated cMyBP-C might trigger different cellular signaling than a pure absence of the protein. We also postulate that heterogeneity of disease severity observed in the human population might be influenced by differences in mutations which produce variably reduced amounts of abnormal protein vs. pure protein reduction. Understanding the pathways to HCM manifestation, and how the cardiomyocyte transition from hyperplasia to hypertrophy influences this course may provide novel insight into this enigmatic disease and allow development of more effective preventative treatments for families known to harbor pathologic mutations.

## Conclusion

A murine HCM model in which cMyBP-C is genetically ablated presents shortly after birth with elevated cardiomyocyte hyperplasia before onset of pathologic cellular hypertrophy evident by PND9. This work implicates cell cycle pathway dysregulation and cardiomyocyte proliferation as early responses that precede the cellular hypertrophic response, and may have implications for the severity of hypertrophy. These data and those from other model systems suggest a relationship between cell cycle and hypertrophic signaling pathways occurring during a critical time period in HCM disease pathogenesis. Awareness of the potential for interplay between cell cycling/proliferation and the hypertrophic signaling pathways provides a new opportunity to expand our understanding of cardiac physiology in both health and disease.

## Author contributions

EF was involved in study design, data collection and analysis, interpretation, writing, and editing. AG was involved in study design, data collection and analysis, writing, interpretation, and editing. AA was involved in data collection and analysis, interpretation, and editing. WdL was involved in study design, data collection and analysis, interpretation, writing and editing. JR was involved in study design, interpretation, writing, and editing.

### Conflict of interest statement

The authors declare that the research was conducted in the absence of any commercial or financial relationships that could be construed as a potential conflict of interest.

## Abbreviations

AMP: adenosine monophosphate
*APC*: anaphase-promoting complex
BW: body weight
*Ccna2*: cyclin A2
*Cdc25c*: cell division cycle 25c
*Cdk*: cyclin dependent kinase
cMyBP-C: cardiac myosin binding protein C (protein)
DCM: dilated cardiomyopathy
E: embryonic day
*Erk 1/2*: extracellular-signal-regulated kinases
GC-A: guanylyl cyclase A
HCM: hypertrophic cardiomyopathy
HW: heart weight
IPA: Ingenuity Pathway Analysis
IVRT: isovolumetric relaxation time
KEGG: The Kyoto Encyclopedia of Genes and Genomes
LVAW: left ventricular anterior wall thickness
LVPW_*s*_: left ventricular posterior wall thickness (during systole)
MHC: myosin heavy chain
*MYBPC3*: human cardiac myosin binding protein C (gene)
*Mybpc3*: mouse cardiac myosin binding protein C (gene)
*Myh6*: beta myosin heavy chain (gene)
*Myh7*: alpha myosin heavy chain (gene)
*Nppa*: atrial natriuretic peptide A
PND: postnatal day
*Prkag2*: protein kinase AMP-activated non-catalytic subunit gamma 2
SCD: sudden cardiac death
WGA: wheat germ agglutinin
WT: wild-type.
