# Supplementary material for: Increased Postnatal Cardiac Hyperplasia Precedes Cardiomyocyte Hypertrophy in a Model of Hypertrophic Cardiomyopathy
Source: Front Physiol. 2017 Jun 14;8:414. doi: 10.3389/fphys.2017.00414 (PMC5470088; doi:10.3389/fphys.2017.00414)
Supplement: Supplementary file 1 [file Table1.DOCX]

| **Supplemental Table I:** Gene Names for Gene IDs/Proteins in IPA Network (Supplementary Figure I) of Focus Genes Differentially Expressed Between WT and cMyBP-C^-/-^ Hearts at PND1 | |
| --- | --- |
| Gene ID/Protein | Gene/Protein* Name |
| APC complex | Anaphase-promoting complex* |
| APC-CDC20 | Anaphase promoting complex-cell division cycle 20 |
| AURKA | Aurora kinase A |
| BUB1 | BUB1 mitotic checkpoint serine/threonine kinase |
| BUB1B | BUB1 mitotic checkpoint serine/threonine kinase B |
| CCNA2 | Cyclin A2 |
| CCNB2 | Cyclin B2 |
| Ccnb1/Gm5593* | Cyclin B1/predicted gene 5593 (mouse) |
| Cdc2 | Cyclin-dependent kinase 1 (mouse) |
| CDC20 | Cell division cycle 20 |
| CDC25C | Cell division cycle 25C |
| Cdk | Cyclin-dependent kinase (mouse) |
| CDK1 | Cyclin-dependent kinase 1 |
| CENPE | Centromere protein E, 312kDa |
| CEP55 | Centrosomal protein 55kDa |
| CCNA1 | Cyclin A* |
| CCNB1 | Cyclin B* |
| FAM83D | Family with sequence similarity 83, member D |
| FBXO5 | F-box protein 5 |
| HMMR | Hyaluronan-mediated motility receptor (RHAMM) |
| ITGB6 | Integrin, beta 6 |
| KCND2 | Potassium voltage-gated channel, Shal-related subfamily, member 2 |
| KIF11 | Kinesin family member 11 |
| KIF22 | Kinesin family member 22 |
| KIF2C | Kinesin family member 2C |
| KNSTRN | Kinetochore-localized astrin/SPAG5 binding protein |
| KPNA/KPNB | Importin alpha/beta* |
| MAPK1/3 | ERK1/2* |
| Mpf | Mesothelin |
| PLK1 | Polo-like kinase 1 |
| PRC1 | Protein regulator of cytokinesis 1 |
| PTTG1 | Pituitary tumor-transforming 1 |
| TPX2 | *TPX2*, microtubule-associated |
| UBE2C | Ubiquitin-conjugating enzyme E2C |
| *protein name | |
