# Supplementary material for: Increased Postnatal Cardiac Hyperplasia Precedes Cardiomyocyte Hypertrophy in a Model of Hypertrophic Cardiomyopathy
Source: Front Physiol. 2017 Jun 14;8:414. doi: 10.3389/fphys.2017.00414 (PMC5470088; doi:10.3389/fphys.2017.00414)
Supplement: Supplementary file 2 [file Table2.DOCX]

| **Supplemental Table II.** “Hypertrophic Cardiomyopathy” is the Top-Scoring Pathway at PND9 in cMyBP-C^-/-^ Hearts | | | | | |
| --- | --- | --- | --- | --- | --- |
| KEGG Pathway | Gene name | Gene ID | Direction | Ratio | p-value |
| Hypertrophic Cardiomyopathy | Myosin binding protein C, cardiac | Mybpc3 | Down | 21.39 | <0.001 |
|  | Myosin, heavy polypeptide 7,  cardiac muscle, beta | Myh7 | Up | 4.09 | 0.044 |
|  | Tropomyosin 2, beta | Tpm2 | Up | 2.87 | <0.001 |
|  | Angiotensin I converting enzyme (peptidyl-dipeptidase A) 1 | Ace | Up | 1.81 | <0.001 |
|  | Solute carrier family 8 (sodium/calcium exchanger), member 1 | Slc8a1 | Up | 1.73 | NS |
|  | Insulin-like growth factor 1 | Igf1 | Up | 1.62 | <0.001 |
|  | Transforming growth factor, beta 2 | Tgfb2 | Up | 1.62 | 7.8E-07 |
|  | Integrin alpha 7 | Itga7 | Down | 1.59 | NS |
|  | Endothelin 3 | Edn3 | Up | 1.53 | 0.001 |
|  | Protein kinase, AMP-activated, beta 2 non-catalytic subunit | Prkab2 | Up | 1.51 | NS |
| Ratio indicates fold difference compared to WT hearts at the same age. NS, not statistically significant. Full list of pathways and genes differentially regulated in cMyBP-C^-/-^ hearts at PND9 according to Genesifter analysis are provided in Supplemental Table III. | | | | | |
