# Supplementary material for: Increased Postnatal Cardiac Hyperplasia Precedes Cardiomyocyte Hypertrophy in a Model of Hypertrophic Cardiomyopathy
Source: Front Physiol. 2017 Jun 14;8:414. doi: 10.3389/fphys.2017.00414 (PMC5470088; doi:10.3389/fphys.2017.00414)
Supplement: Supplementary file 3 [file Table3.DOCX]

| **Supplemental Table III:** KEGG Pathways of Genes Differentially Regulated Between WT and cMyBP-C^-/-^  Hearts at PND9 | | | | | |
| --- | --- | --- | --- | --- | --- |
| **KEGG Pathway** | **Ratio** | **Direction** | **Gene ID** | **Gene name** | **Cardiovascular disease states identified by Malacards** |
| **Hypertrophic Cardiomyopathy** | 21.39 | Down | Mybpc3 | Myosin binding protein C, cardiac | Coronary Heart Disease; Familial Hypertrophic Cardiomyopathy; Hypertension; Myocardial infarction |
|  | 4.09 | Up | Myh7 | Myosin, heavy polypeptide 7, cardiac muscle, beta | Dilated cardiomyopathy; Hypertrophic Cardiomyopathy; Myocardial infarction |
|  | 2.87 | Up | Tpm2 | Tropomyosin 2, beta | Familial Hypertrophic Cardiomyopathy |
|  | 1.81 | Up | Ace | Angiotensin I converting enzyme (peptidyl-dipeptidase A) 1 | Coronary Heart Disease; Familial Hypertrophic Cardiomyopathy; Hypertension; Myocardial infarction |
|  | 1.73 | Up | Slc8a1 | Solute carrier family 8 (sodium/calcium exchanger), member 1 | Cardiac hypertrophy; Hypertension |
|  | 1.62 | Up | Igf1 | Insulin-like growth factor 1 | Coronary Heart Disease; Familial Hypertrophic Cardiomyopathy; Hypertension; Myocardial infarction |
|  | 1.62 | Up | Tgfb2 | Transforming growth factor, beta 2 | Atherosclerosis; Dilated cardiomyopathy; Hypertension |
|  | 1.59 | Down | Itga7 | Integrin alpha 7 | Atherosclerosis; Dilated cardiomyopathy; Hypertension; Hypertrophic Cardiomyopathy; Myocardial infarction |
|  | 1.53 | Up | Edn3 | Endothelin 3 | Hypertension |
|  | 1.51 | Up | Prkab2 | Protein kinase, AMP-activated, beta 2 non-catalytic subunit | Atherosclerosis; Atrial septal defect |
| **Protein digestion and absorption** | 2.2 | Down | Mme | Membrane metallo endopeptidase | Atherosclerosis; Congestive heart failure; Hypertension; Myocardial infarction |
|  | 2.16 | Down | Atp1a2 | ATPase, Na+/K+ transporting, alpha 2 polypeptide | Hypertension; Vascular disease |
|  | 2.03 | Up | Eln | Elastin | Atherosclerosis; Ischemic heart disease; Pulmonary valve stenosis |
|  | 1.97 | Up | Kcne3 | Potassium voltage-gated channel, Isk-related subfamily, gene 3 | Atrial fibrillation; Long QT syndrome |
|  | 1.73 | Up | Slc8a1 | Solute carrier family 8 (sodium/calcium exchanger), member 1 | Hypertension |
|  | 1.68 | Down | Dpp4 | Dipeptidylpeptidase 4 | Atherosclerosis |
|  | 1.55 | Up | Slc9a3 | Solute carrier family 9 (sodium/hydrogen exchanger), member 3 | Hypertension |
| **Toxoplasmosis** | 1.97 | Up | Pla2g4a | Phospholipase A2, group IVA (cytosolic, calcium-dependent) | Atherosclerosis; Hypertension |
|  | 1.79 | Down | Pla2g4e | CDNA clone IMAGE:9053568 | Atherosclerosis |
|  | 1.75 | Up | Tlr4 | Toll-like receptor 4 | Atherosclerosis; Cardiomyopathy; Hypertension; Myocardial infarction |
|  | 1.52 | Down | Map2k6 | MAP Kinase Kinase | Cardiomyopathy; Myocardial infarction |
| **Focal adhesion** | 7.21 | Up | Spp1 | Osteopontin | Atherosclerosis; Dilated cardiomyopathy; Hypertension; Myocardial infarction |
|  | 3.45 | Up | Thbs4 | Thrombospondin 4 | Atherosclerosis; Cardiomyopathy; Coronary heart disease; Myocardial infarction |
|  | 2.58 | Down | Vtn | Vitronectin | Atherosclerosis; Myocardial infarction |
|  | 2.36 | Up | Tnc | Tenascin C | Atherosclerosis; Dilated cardiomyopathy; Hypertension; Myocardial infarction |
|  | 1.54 | Up | Myl9 | Myosin, light polypeptide 9 | Familial Hypertrophic Cardiomyopathy |
|  | 1.5 | Up | Thbs1 | Thrombospondin 1 | Atherosclerosis; Cardiomyopathy; Coronary heart disease; Myocardial infarction |
| **Phagosome** | 1.72 | Up | C3 | Complement component 3 | Atherosclerosis; Dilated cardiomyopathy; Hypertension; Myocardial infarction |
|  | 1.5 | Up | Ctss | Cathepsin S | Atherosclerosis; Coronary heart disease |
| **Vascular smooth muscle contraction** | 6.06 | Up | Nppa | Natriuretic peptide precursor type A | Atherosclerosis; Dilated cardiomyopathy; Hypertension; Hypertrophic Cardiomyopathy; Myocardial infarction |
|  | 3.2 | Down | Pla2g5 | Phospholipase A2, group V | Atherosclerosis |
|  | 1.97 | Up | Pla2g4a | Phospholipase A2, group IVA (cytosolic, calcium-dependent) | Atherosclerosis; Hypertension |
|  | 1.78 | Up | Nppb | Natriuretic peptide precursor type B | Atherosclerosis; Dilated cardiomyopathy; Hypertension; Hypertrophic Cardiomyopathy; Myocardial infarction |
|  | 1.69 | Down | Adra1b | Adrenergic receptor, alpha 1b | Hypertension |
| **Chagas disease (American trypanosomiasis)** | 1.98 | Up | Serpine1 | Serine (or cysteine) peptidase inhibitor, clade E, member 1 | Atherosclerosis; Coronary heart disease; Dilated cardiomyopathy; Hypertension; Myocardial infarction |
|  | 1.57 | Up | Ccl2 | Strain NOD/LtJ small inducible cytokine A2 precursor | Atherosclerosis; Dilated cardiomyopathy; Hypertension; Hypertrophic Cardiomyopathy; Myocardial infarction |
| **PPAR signaling pathway** | 3.71 | Down | Rxrg | Retinoid X receptor gamma | Atherosclerosis; Coronary heart disease |
|  | 1.89 | Up | Angptl4 | Angiopoietin-like 4 | Coronary heart disease; Hypertension |
|  | 1.62 | Down | Pltp | Phospholipid transfer protein | Atherosclerosis; Coronary heart disease |
|  | 1.52 | Down | Aqp7 | Aquaporin 7 | Atherosclerosis |
| **TGF-beta signaling pathway** | 2.39 | Up | Inhba | Inhibin beta-A (Inhba), mRNA | Hypertension; Myocardial infarction |
| **p53 signaling pathway** | 2.31 | Up | Igfbp3 | Insulin-like growth factor binding protein 3 | Atherosclerosis; Coronary heart disease; Hypertension; Hypertrophic Cardiomyopathy; Myocardial infarction |
