# Supplementary material for: Increased Postnatal Cardiac Hyperplasia Precedes Cardiomyocyte Hypertrophy in a Model of Hypertrophic Cardiomyopathy
Source: Front Physiol. 2017 Jun 14;8:414. doi: 10.3389/fphys.2017.00414 (PMC5470088; doi:10.3389/fphys.2017.00414)
Supplement: Supplementary file 4 [file Table4.DOCX]

| **Supplemental Table IV:** Number of pups/hearts per day/genotype for analysis of pup weight and HW to BW ratio (Figure 3). | | | |
| --- | --- | --- | --- |
|  | WT | +/- | -/- |
| (expected) | (25%) | (50%) | (25%) |
| E18.5 | 9 | 28 | 15 |
| PND0 | 17 | 43 | 18 |
| PND1 | 21 | 41 | 13 |
| PND2 | 24 | 49 | 25 |
| PND9 | 32 | 47 | 13 |
