# Supplementary material for: Increased Postnatal Cardiac Hyperplasia Precedes Cardiomyocyte Hypertrophy in a Model of Hypertrophic Cardiomyopathy
Source: Front Physiol. 2017 Jun 14;8:414. doi: 10.3389/fphys.2017.00414 (PMC5470088; doi:10.3389/fphys.2017.00414)
Supplement: Supplementary file 5 [file Table5.DOCX]

| **Supplemental Table V:** Cardiomyocyte Size at PND2 and PND9 | | | | | | | |
| --- | --- | --- | --- | --- | --- | --- | --- |
|  | PND2 | | |  | PND9 | | |
|  | WT | +/- | -/- |  | WT | +/- | -/- |
| length (µm) | 48.96 ± 10.34 | 52.00 ± 11.81 | 47.88 ± 10.95† |  | 60.94 ± 9.79 | 64.41 ± 11.10* | 69.44 ± 14.16*† |
| width (µm) | 6.82 ± 1.33 | 7.14 ± 1.55 | 7.03 ± 1.55 |  | 9.36 ± 1.58 | 9.65 ± 1.56 | 9.85 ± 1.98 |
| area (L x W; µm^2^) | 338.22 ± 118.61 | 377.27 ± 147.34 | 341.21 ± 124.19 |  | 574.81 ± 147.94 | 627.09 ± 159.63 | 695.20 ± 234.48* |
| Values are means ±SD. L, length; W, width; WT, wild type; +/-, cMyBP-C^+/-^; -/-, cMyBP-C^-/-^. n=3 hearts/genotype, n=50 cells/heart (PND9) and 100cells/heart (PND2). *p<0.05 vs WT, † vs +/-. | | | | | | | |
