# Supplementary material for: Increased Postnatal Cardiac Hyperplasia Precedes Cardiomyocyte Hypertrophy in a Model of Hypertrophic Cardiomyopathy
Source: Front Physiol. 2017 Jun 14;8:414. doi: 10.3389/fphys.2017.00414 (PMC5470088; doi:10.3389/fphys.2017.00414)
Supplement: Supplementary file 6 [file Table6.DOCX]

| **Supplemental Table VI:** Genes Upregulated in Microarray of PND1 cMyBP-C^-/-^ Hearts, and which are Thought to be Involved in Cytokinetic Processes of Assembly of Contractile Ring, Cleavage Furrow or in Abcission | | | | |
| --- | --- | --- | --- | --- |
| **Gene name** | **Gene ID** | **Fold change** | | **References** |
| Epithelial cell transforming 2 | Ect2 | | 1.6 | ^1-5^ |
| Anillin | Anln | | 1.6 | ^1, 2, 4, 6, 7^ |
| Rac GTPase activating protein | MgcRacGAP, Cyk-4 | | 1.6 | ^1, 3-5^ |
| Annexin A2 | Anxa2 | | 1.5 | ^3^ |
| centrosomal protein 55 | Cep55 | | 1.6 | ^8^ |

**Supplemental References**

1. Barr FA and Gruneberg U. Cytokinesis: placing and making the final cut. *Cell*. 2007;131:847-60.

2. Miller AL. The contractile ring. *Current biology : CB*. 2011;21:R976-8.

3. Benaud C, Le Dez G, Mironov S, Galli F, Reboutier D and Prigent C. Annexin A2 is required for the early steps of cytokinesis. *EMBO reports*. 2015;16:481-9.

4. Normand G and King RW. Understanding Cytokinesis Failure. *Advances in experimental medicine and biology*. 2010;676:27-55.

5. Glotzer M. The molecular requirements for cytokinesis. *Science (New York, NY)*. 2005;307:1735-9.

6. Piekny AJ and Glotzer M. Anillin is a scaffold protein that links RhoA, actin, and myosin during cytokinesis. *Current biology : CB*. 2008;18:30-6.

7. Sun L, Guan R, Lee IJ, Liu Y, Chen M, Wang J, Wu JQ and Chen Z. Mechanistic insights into the anchorage of the contractile ring by anillin and Mid1. *Dev Cell*. 2015;33:413-26.

8. Carlton JG and Martin-Serrano J. Parallels between cytokinesis and retroviral budding: a role for the ESCRT machinery. *Science (New York, NY)*. 2007;316:1908-12.
