# Supplementary material for: Increased Postnatal Cardiac Hyperplasia Precedes Cardiomyocyte Hypertrophy in a Model of Hypertrophic Cardiomyopathy
Source: Front Physiol. 2017 Jun 14;8:414. doi: 10.3389/fphys.2017.00414 (PMC5470088; doi:10.3389/fphys.2017.00414)
Supplement: Supplementary file 7 [file DataSheet1.DOCX]

**Supplemental Materials**

**Methods**

**Routine histology**

PND1 and PND9 mouse hearts harvested as described were fixed in Dent’s fixative (20% DMSO/80% MeOH) at 4°C for 48 hours. The samples were subsequently transferred to 100% MeOH and stored at −20°C for at least 48 hours. Fixed hearts were transferred directly to xylenes and allowed to equilibrate for 1 hour before being placed in heated paraffin and maintained at 65°C for 120 minutes. They were subsequently embedded in fresh heated paraffin in disposable plastic molds and allowed to cool before being sectioned at 8 µm on an RM2165 microtome (Leica), mounted on charged glass microscope slides (Superfrost Plus; Thermo Fisher Scientific), and allowed to dry overnight at 35°C on a flattening plate. For histological assessment, slides were incubated at 68°C for 1 hour, submerged twice in xylenes for 2 minutes each, and rehydrated through EtOH series to water, and then stained with either hematoxylin and eosin (H&E) or Masson’s Trichrome or Elastic Trichrome stains (Sigma) to highlight elastic fibers and collagen, or by picrosirius red stain (Polysciences, Inc.) to highlight interstitial fibrosis. After staining, sections were coverslipped using Micromount slide-mounting fluid (Surgipath).

**Preparation of mice for transthoracic** **echocardiography** **on WT and cMyBPC^−/−^ mice**

PND9 mice were anaesthetized using inhaled 1% isoflurane, with flow titrated to minimize bradycardia and maintain a target heart rate of 450 ±50 beats per minute. Conscious PND1 and anesthetized PND9 mice were taped in a left lateral decubitus position on a warmed platform, and chest hair removed with a topical depilatory agent prior to ultrasound imaging.

**RNA isolation and RT-qPCR.**

RNA was isolated from hearts of each age and genotype as follows. The tissue was homogenized using Trizol reagent (Invitrogen) and an appropriate amount of chloroform (Sigma-Aldrich) was added, according to the manufacturer’s instructions. After mixing, incubation, and centrifugation, the aqueous phase containing RNA was collected and treated with RNase-free DNase I (Qiagen) for 30 minutes before being treated using the Mini Total RNA Kit (Blood/Cultured Cells; IBI Scientific). 500 ng total RNA was reverse transcribed into first-strand cDNA with the iScript Reverse Transcription Supermix for RT-qPCR kit (Bio-Rad) following manufacturer’s protocols. 10 ng of the resulting cDNA sample was used for qRT‑PCR analysis. Primers for assayed genes and appropriate controls were arrayed in MicroAmp Optical 96-well Reaction Plates (Applied Biosystems) and the PCR performed using the TaqMan Gene Expression Master Mix (Applied Biosystems). Real-time monitoring of TaqMan fluorescence was performed on the Stratagene Mx3005P qPCR system (Agilent Technologies). An initial activation step of 95°C for 10 minutes was followed by 40 cycles of 15 seconds denaturation and 60 seconds annealing/extension. Data was analyzed using Excel, using the ΔΔCT method, with *Gapdh*/*Actb* used as housekeeping control genes. TaqMan assay IDs used were: Gapdh (Mm99999915_g1), Actb (Mm00607939_s1), Nppa (Mm01255748_g1), Myh6 (Mm00440359_m1), Myh7 (Mm00600555_m1), Ccna2 (Mm00438063_m1), and Cdc25c (Mm00486872).

**Immunohistochemistry**

For IHC on paraffin-embedded hearts, slides with coronal heart sections were rehydrated through ethanol series to water, and rinsed twice in 1X PBS prior to IHC. Both paraffin and cryogenic sections were blocked for 1 h in 1X PBS containing 5% (vol/vol) sheep serum, 2 mg/ml bovine serum albumin, and 0.1% (vol/vol) Tween 20 and then incubated with primary antibodies in blocking buffer for 1-2 hours in a humidity chamber at room temperature. Between incubations, slides were rinsed 2 × 5 minutes in 1X PBS containing 0.1% (vol/vol) Tween 20 and paraffin sections were re-blocked in blocking buffer for 5 minutes. Sections were incubated with secondary antibody in blocking buffer for 1 hour. As a control, similar sections were incubated with secondary antibodies only. Following labeling, sections were rinsed 2 x 5 minutes in 1X PBS containing 0.1% (vol/vol) Tween 20, then 1 x 5 minutes in water, and coverslipped using warmed ProLong Gold Antifade Reagent (Invitrogen) with 4′,6-diamidino-2-phenylindole (DAPI) to label nuclei.

**Cryogenic preservation of hearts, cryosectioning and IHC**

Hearts used for cryosectioning were rinsed in 1X PBS after extraction and transferred to a solution of filtered 30% sucrose in 1X PBS for at least one hour, until the heart equilibrated and sank. Hearts were then transferred into a solution containing a 1:1 mixture of optimal cutting temperature (OCT) compound and 30% sucrose in PBS for 1 hour. Hearts were positioned apex down on cold cryomolds resting on dry ice. Cryomolds were then filled with OCT compound and care was taken to keep hearts correctly positioned until frozen. Hearts in OCT were then stored at -80˚C until sectioned. After genotyping, WT and cMyBP-C^-/-^ hearts were cryosectioned (CRYO 03/5800, Tissue-Tek) in a transverse orientation at 6 µm and mounted on charged glass slides (TruBond 380). Once sections were completely dry, they were fixed in pre-cooled, 100% acetone at 4˚C for 15 minutes and then re-dried. Sections were rehydrated by incubating slides in 1X PBS for 3 x 5 minutes. Sections were blocked for 1 hour in 1X PBS containing 5% (vol/vol) normal goat serum, 2 mg/ml bovine serum albumin, and 0.1% (vol/vol) Tween 20. Sections were then incubated for 1 hour with 1:1000 anti-α-actinin mouse monocolonal antibody (Sigma, A7811) to label cardiomyocytes. All antibody incubations were performed in a humidity chamber at room temperature. Slides were then washed 3 x 5 minutes in 1X PBS containing 0.1% (vol/vol) Tween 20 and then incubated with the secondary antibody AlexaFluor 568 goat anti-mouse IgG_1_ (Molecular Probes) at 1:250 for 1 hour. Dilutions of antibodies were made in blocking buffer. Sections were rinsed 3 x 5 minutes in 1X PBS containing 0.1% (vol/vol) Tween 20 and then incubated for 10 minutes with 5 µg/ml wheat germ agglutinin (WGA) - Alexa 647 (Molecular Probes) (for the determination of cross‑sectional area [CSA]) in 1X PBS. Following labeling, sections were rinsed 3 x 5 minutes in 1X PBS then 1 x 5 minutes in water, and coverslipped using ProLong Gold Antifade Reagent (Invitrogen) with 4′,6-diamidino-2-phenylindole (DAPI) to label nuclei.

**Measurements of cell cycling in cryogenic heart sections**

For analysis of cell cycling from cryogenic heart sections, measurements were taken from 3 hearts per genotype/age, with sampling from 3 cryosections per heart and 3 fields per section. The investigator performing the measurements and counts was blind to the genotype of all heart sections.

Cardiomyocyte cell cycling on PND2 heart cryosections (Supplemental Figure IV) was determined by labeling with anti-α-actinin at 1:1000 (mouse monoclonal; Sigma, A7811), anti-Ki-67, a marker of cell cycling, at 1:200 (rabbit polyclonal antibody; Abcam, ab15580), and DAPI. Within each field, all DAPI‑stained nuclei belonging to cardiomyocytes (confirmed by α-actinin labeling) were counted, followed by a count of DAPI-stained cardiomyocyte nuclei that were also positive for Ki-67 labeling. The fractions of Ki-67-positive nuclei per total cardiomyocyte nuclei were calculated for each field and averaged per heart. The averages for each heart were compared between genotypes within each age. All calculations were analyzed using Students’ t-test.

**Imaging**

Imaging was performed using the Nikon Eclipse 90i advanced motorized research photomicroscope equipped with the Intensilight C-HGFI fluorescence source and DS-QiMc monochrome and DS-Fi1 color cameras using Pan Fluor 4x/0.13 air, Pan Fluor 10x/0.30 air and S Fluor 40×/1.30 oil objectives, and NIS‑Elements imaging software (version 4.0; Nikon).
