# Supplementary material for: Increased Postnatal Cardiac Hyperplasia Precedes Cardiomyocyte Hypertrophy in a Model of Hypertrophic Cardiomyopathy
Source: Front Physiol. 2017 Jun 14;8:414. doi: 10.3389/fphys.2017.00414 (PMC5470088; doi:10.3389/fphys.2017.00414)
Supplement: Supplementary file 8 [file Image1.PDF]

SUPPLEMENTAL FIGURES

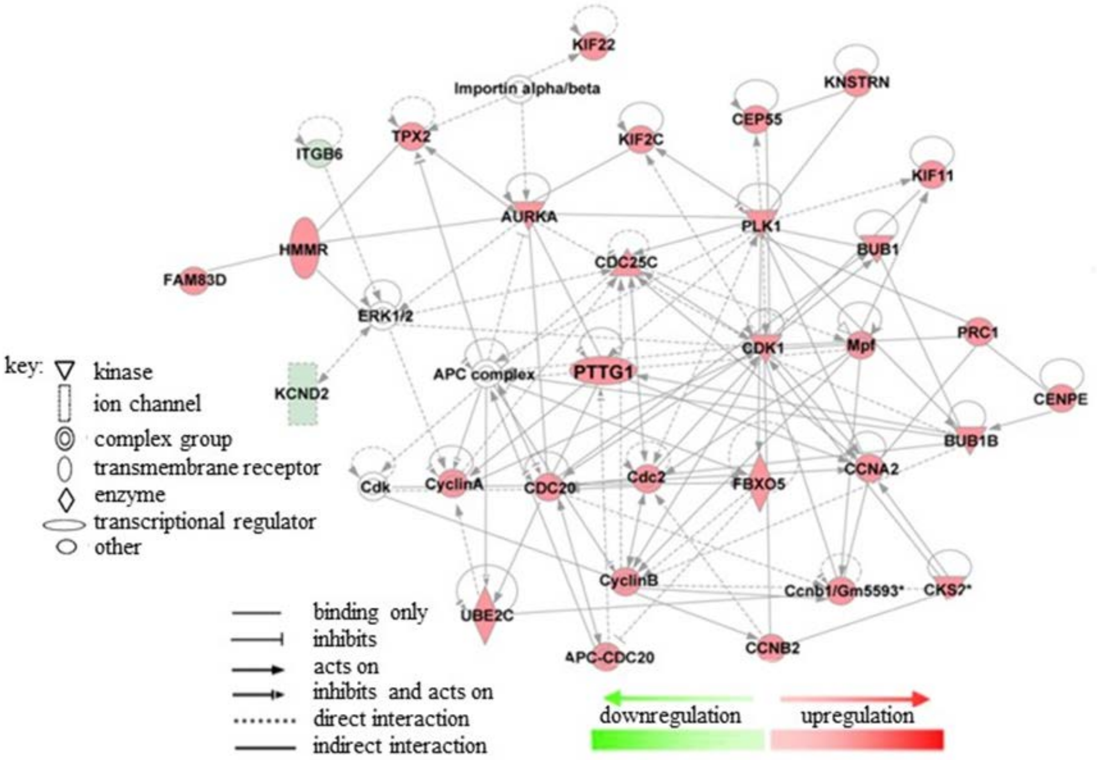

Supplemental Figure I. Ingenuity IPA network assembled from 26 focus genes that were differentially expressed in wild type versus cMyBP-C$^{-/-}$ hearts at PND1. Many of the genes listed play roles in cell cycling (see text for details). Input genes are depicted as nodes. Uncolored nodes were also included, indicating a strong biological relevance to the network based upon evidence from the IPA knowledge base. Online Table I provides gene names for each gene ID presented in the figure.
