# Supplementary material for: Increased Postnatal Cardiac Hyperplasia Precedes Cardiomyocyte Hypertrophy in a Model of Hypertrophic Cardiomyopathy
Source: Front Physiol. 2017 Jun 14;8:414. doi: 10.3389/fphys.2017.00414 (PMC5470088; doi:10.3389/fphys.2017.00414)
Supplement: Supplementary file 9 [file Image2.PDF]

SUPPLEMENTAL FIGURES

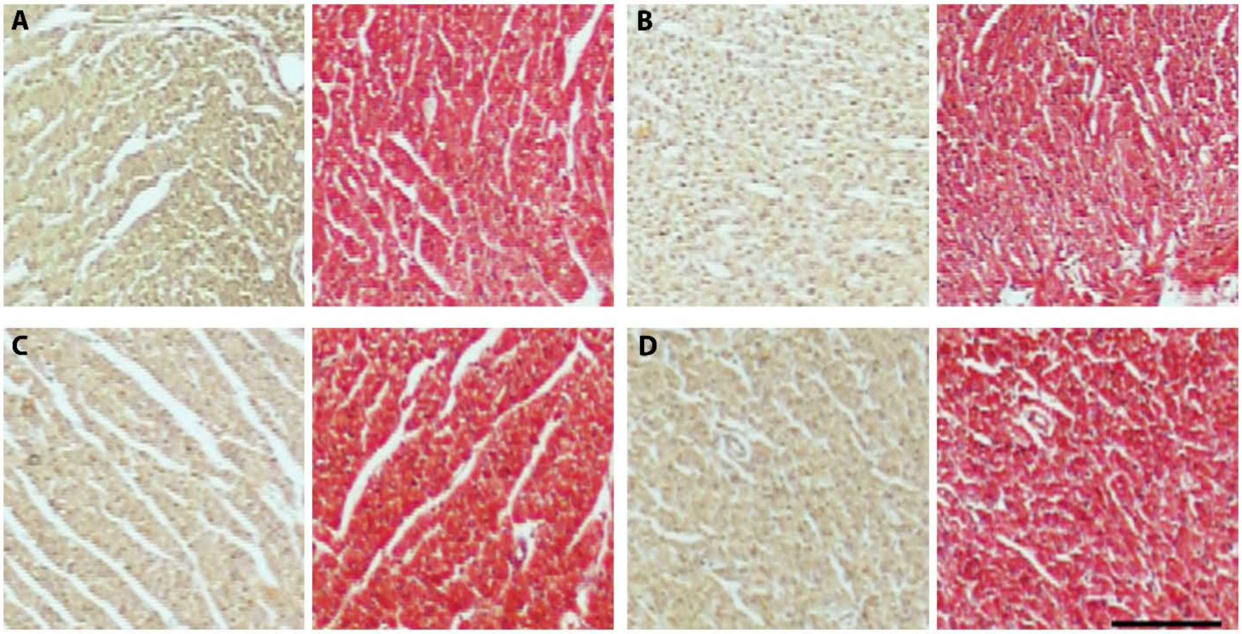

Supplemental Figure II. Sections of hearts from wildtype (A, C) and cMyBP-C$^{-/-}$ (B, D) mice at PND1 (A, B) and PND9 (C, D), stained with elastic trichrome (left image in each panel) and Masson's trichrome (right image in each panel). Representative areas of each heart are shown. Scale bar: 100 µm.
