# Supplementary material for: Increased Postnatal Cardiac Hyperplasia Precedes Cardiomyocyte Hypertrophy in a Model of Hypertrophic Cardiomyopathy
Source: Front Physiol. 2017 Jun 14;8:414. doi: 10.3389/fphys.2017.00414 (PMC5470088; doi:10.3389/fphys.2017.00414)
Supplement: Supplementary file 10 [file Image3.PDF]

SUPPLEMENTAL FIGURES

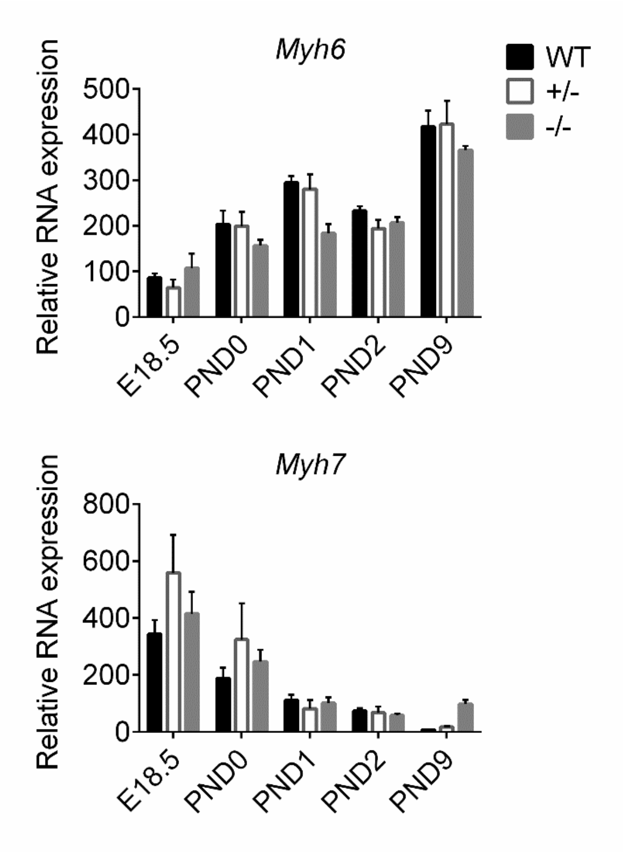

Supplemental Figure III. Expression of *Myh6* and *Myh7* in WT, cMyBP-C$^{+/-}$ (+/-), and cMyBP-C$^{-/-}$ (-/-) from E18.5 to PND9 relative to the average of 2 housekeeping genes, *Gapdh* and *ß-actin.* n ≥5 hearts/group.
