# Supplementary material for: Increased Postnatal Cardiac Hyperplasia Precedes Cardiomyocyte Hypertrophy in a Model of Hypertrophic Cardiomyopathy
Source: Front Physiol. 2017 Jun 14;8:414. doi: 10.3389/fphys.2017.00414 (PMC5470088; doi:10.3389/fphys.2017.00414)
Supplement: Supplementary file 11 [file Image4.pdf]

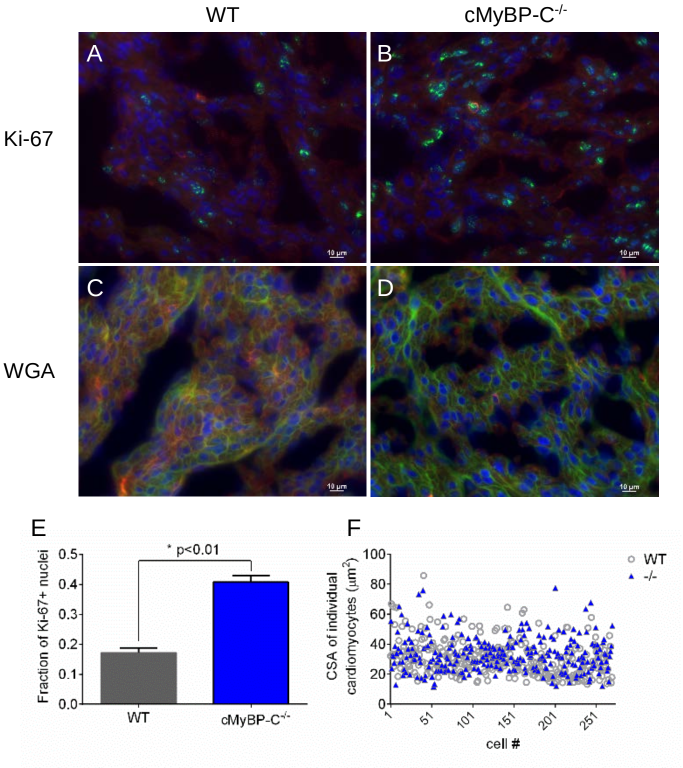

Supplementary Figure IV. Elevated cell cycling without hypertrophy in cMyBP-C$^{-/-}$ hearts at PND2. A-D: Representative sections of hearts of WT (A, C) and cMyBP-C$^{-/-}$ (B, D) mice at PND2. (A, B) Sections are labeled with α-actinin (red), a muscle-specific marker, Ki-67 (pink) to highlight cells within the cell cycle, and counterstained with DAPI (blue) to highlight nuclei. (C, D) Sections labeled with α-actinin (red) and wheat germ agglutinin (WGA; pink) to highlight cell borders, and counterstained with DAPI (blue). Quantification of Ki-67-positive cardiomyocytes (E) and cardiomyocyte cross-sectional areas of individual myocytes (CSA; F) for WT and cMyBP-C$^{-/-}$ (-/-) PND2 heart cross sections, as shown in A-H. n=3 hearts, 3 sections per heart from each genotype. Means ±SE are reported, *$p<0.05$.
